# Target Control in Logical Models Using the Domain of Influence of Nodes

**DOI:** 10.3389/fphys.2018.00454

**Published:** 2018-05-08

**Authors:** Gang Yang, Jorge Gómez Tejeda Zañudo, Réka Albert

**Affiliations:** ^1^Department of Physics, Pennsylvania State University, University Park, PA, United States; ^2^Department of Medical Oncology, Dana-Farber Cancer Institute, Boston, MA, United States; ^3^Eli and Edythe L. Broad Institute of MIT and Harvard, Cambridge, MA, United States; ^4^Department of Biology, Pennsylvania State University, University Park, PA, United States

**Keywords:** target control, Boolean network, biological network, domain of influence, logical modeling, network dynamics

## Abstract

Dynamical models of biomolecular networks are successfully used to understand the mechanisms underlying complex diseases and to design therapeutic strategies. Network control and its special case of target control, is a promising avenue toward developing disease therapies. In target control it is assumed that a small subset of nodes is most relevant to the system's state and the goal is to drive the target nodes into their desired states. An example of target control would be driving a cell to commit to apoptosis (programmed cell death). From the experimental perspective, gene knockout, pharmacological inhibition of proteins, and providing sustained external signals are among practical intervention techniques. We identify methodologies to use the stabilizing effect of sustained interventions for target control in Boolean network models of biomolecular networks. Specifically, we define the domain of influence (DOI) of a node (in a certain state) to be the nodes (and their corresponding states) that will be ultimately stabilized by the sustained state of this node regardless of the initial state of the system. We also define the related concept of the logical domain of influence (LDOI) of a node, and develop an algorithm for its identification using an auxiliary network that incorporates the regulatory logic. This way a solution to the target control problem is a set of nodes whose DOI can cover the desired target node states. We perform greedy randomized adaptive search in node state space to find such solutions. We apply our strategy to *in silico* biological network models of real systems to demonstrate its effectiveness.

## 1. Introduction

In cellular systems various molecular species, such as DNA, RNA, proteins and small molecules, interact in diverse ways. The totality of these interactions gives rise to cellular functions. The relationship between molecular interacting systems and cellular functions is studied in the new emerging field of systems biology (Alon, 2006; Palsson, 2006). A promising systems biology methodology is to represent the molecular interacting system as a network, construct a dynamic model of the information propagation on this network, and identify the cellular functions with long-term behaviors of the dynamic model (Palsson, 2006; Newman, 2010; Wang et al., 2012; Barabási and Pósfai, 2016). Various types of dynamical models of biological networks have been built to integrate related experimental results and to reveal the underlying mechanisms of complex diseases such as cancers, and predict beneficial interventions. Quantitative, mechanistic models, generally using systems of ordinary or partial differential equations, can be highly accurate and provide quantitative information (e.g., response time to a signal, or fold-changes of protein concentrations) (Tyson et al., 2003, 2011; Alon, 2006; Iyengar et al., 2012). These models' widespread use is limited by the scarcity of high-quality quantitative data, such as kinetic and temporal information about individual nodes in the network. Logical models using discrete variables, such as Boolean network models, have the advantage of being scalable and not requiring detailed knowledge of kinetic parameters (Morris et al., 2010; Wynn et al., 2012; Saadatpour and Albert, 2013; Laubenbacher et al., 2014; Abou-Jaoudé et al., 2016; Bloomingdale et al., 2018; Zañudo et al., 2018). An abundance of recent literature has shown that logical models can capture the emergent behaviors of real biological systems, they can generate predictions that are validated by follow-up experiments and they can predict successful intervention strategies (Li et al., 2004; Espinosa-Soto et al., 2004; Mendoza, 2006; Saez-Rodriguez et al., 2007; Naldi et al., 2010; Miskov-Zivanov et al., 2013; Steinway et al., 2015; Albert et al., 2017; Gómez Tejeda Zañudo et al., 2017). For example, logical models of signaling networks that underlie hallmarks of cancer identified the key mechanisms that yield cancer phenotypes and predicted therapeutic interventions that disrupt these phenotypes; many of these predictions were validated experimentally (Grieco et al., 2013; Cohen et al., 2015; Méndez-López et al., 2017; Khan et al., 2017; Kim et al., 2017). Discrete and quantitative models are often consistent in capturing the response repertoire of biological networks (e.g., their potential bistability or response to perturbations) (Kraeutler et al., 2010; Steinway et al., 2016).

Analysis of a logical model entails the determination of the attractors (long-term behaviors) of the system and of the initial states that converge into each attractor (the basins of attraction). Among other uses, this information is used to identify therapeutic interventions as interventions that make a disease attractor unreachable or unstable (Samaga et al., 2010; Abou-Jaoudé et al., 2015; Kim et al., 2017). Attractor identification can be accomplished by simulations of the system's trajectories, determination of all allowed state transitions, or by formal methods such as model checking (Klarner and Siebert, 2015; Abou-Jaoudé et al., 2016), process hitting (Paulevé et al., 2012), or Groebner bases (Laubenbacher et al., 2014). The state space of logical models is finite, but its size scales exponentially with the number of nodes, and thus its full mapping is impossible for systems with many elements. Methods that determine the attractor repertoire of logical models without state space exploration provide a desirable complement to dynamical methods. For example, it was shown that the presence or absence of positive and negative feedback loops in the interaction network puts bounds on the type and number of attractors; e.g., a necessary condition of multistability is the existence of a positive feedback loop (Thomas and D'Ari, 1990; Paulevé and Richard, 2012).

Network control has recently become a popular research topic as it reflects our interest to not only understand an interacting system, but also intervene in it and modify its outcomes (Motter, 2015; Liu and Barabási, 2016). Network control is a broad subject; different underlying models, different control goals and different possible interventions can be considered (Liu and Barabási, 2016). Various control strategies have been designed for both continuous dynamical systems (Liu et al., 2011; Cornelius et al., 2013; Mochizuki et al., 2013; Wells et al., 2015; Wang et al., 2016; Zañudo et al., 2017) and discrete ones (Murrugarra and Dimitrova, 2015; Zañudo and Albert, 2015; Murrugarra et al., 2016; Yang et al., 2016). Of particular interest are the methods that do not require knowledge of the detailed dynamics and parameters of the system, but instead are largely based on the structure of the interaction network and generic assumptions about the functional form of the dependences among variables. In electric circuits modeled by a system of linear ordinary differential equations, it is possible to use graph theoretical methods to identify the set of nodes whose external control can drive the system to any state from any initial condition (Lin, 1974; Liu et al., 2011). For systems with non-linear dynamics, attractor control, that is, to drive the system to one of its natural attractors from any initial condition, has been achieved in several modeling frameworks. Among these, two methods are based on the control of feedback loops: feedback vertex control for ordinary differential equation models (Mochizuki et al., 2013) and stable motif control for logic (Boolean) models (Zañudo and Albert, 2015). However, in biological systems it is not necessary and often not practical to control every component of the system. A more realistic problem is target control, where we assume that the state of the system is characterized by a subset of components and the control goal is to drive these components into desired states. The target control problem has been considered in systems with linear dynamics by Gao et al. (2014), who identified sets of nodes which, if put under suitable (potentially time-varying) external control, drive the target nodes into the desired state.

Despite recent progress in molecular biology, quantitatively manipulating the level of a chemical species is still a challenging problem for experimentalists. Thus any control strategy involving applying time-dependent, variable signals to a target is hard to implement in real systems. However, gene knockout, pharmacological inhibition of proteins and providing sustained external signals have been robustly implemented and demonstrated to be effective intervention strategies (Hopkins and Groom, 2002; Nicholl, 2008; Shalem et al., 2014). Thus we choose our intervention options to be maintaining a sustained state (either absence or abundant activity) in order to make the solution more practical. The effect of such interventions to achieve target control in Boolean network models was previously considered by Klamt et al. (2006) and Samaga et al. (2010). Klamt et al. used the interaction network and regulatory logic to identify the effect of interventions, and determined minimal intervention sets by systematic consideration of all single interventions and combinations of interventions. Samaga et al. made the search for interventions more efficient by using filtering strategies based on the interaction network (e.g., if a candidate intervention source has only negative paths to a target node, then an activating intervention of the source is not useful for activation of the target node) and by grouping equivalent interventions (e.g., if activating a node is sufficient for activating a direct neighbor, then these interventions are equivalent and only one of them needs to be considered).

Here we propose an alternative and complementary intervention prediction method that uses heuristics based on the system-wide influence of the intervention due both to the connectivity and regulatory logic of the modeled system. Specifically, we base these heuristics on each node's domain of influence (DOI), which identifies which other nodes will adopt a fixed state following an intervention that maintains a sustained state of this node, regardless of the system's initial state. While in general determining the DOI of a node requires exploration of the state space, here we introduce the related concept of logical domain of influence (LDOI) of a node, which can be determined based on the interaction network and the regulatory logic. Specifically, the LDOI is defined on the so-called expanded network introduced in (Albert and Othmer, 2003; Wang and Albert, 2011), which is similar in spirit to a logical interaction hypergraph (Klamt et al., 2006). We use the size and internal consistency of the logic domain of influence (LDOI) to inform a greedy randomized adaptive search to identify the sets of nodes whose DOI can cover the desired target node state (combination).

In the following, we give background information on the Boolean modeling framework and relevant previously-developed concepts such as the expanded network and stable motifs. Then we define the DOI and LDOI of a node or multiple nodes and analyze their properties, such as their internal consistency (or lack thereof) and relationship to dynamic attractors. We then define our target control problem and describe our DOI-based target control strategy using greedy randomized adaptive search in node state space. We finally illustrate the effectiveness of our target control strategy in random ensembles and four *in silico* biological network models.

## 2. Materials and methods

### 2.1. Background on boolean network models of biological systems

A dynamical model of a biological system starts with the construction of a network (graph) consisting of nodes (also called vertices) that represent the system's elements and edges that specify the pairwise relationships between nodes. In biological networks at the molecular level, nodes are molecular species such as small molecules, RNA, protein, and edges indicate interactions and regulatory relationships. In discrete dynamic (also called logical) models, each node *i* is characterized by a discrete state variable σ_*i*_, and the vector (σ_1_, ⋯, σ_*n*_) represents the state of the system (Morris et al., 2010; Wynn et al., 2012; Saadatpour and Albert, 2013; Laubenbacher et al., 2014; Abou-Jaoudé et al., 2016; Bloomingdale et al., 2018; Zañudo et al., 2018). The state of the system can be followed in continuous time or at discrete time intervals. In discrete time models, the activity of each node σ_*i*_ is described by a regulatory function σ_*i*_(*t* + τ_*i*_) = *f*_*i*_(σ_*i*_1__(*t*), ⋯, σ_*i*_*k*__(*t*)), where *i*_*1*_, ⋯, *i*_*k*_ are the regulating nodes of *i* and τ_*i*_ is a discrete time delay. The regulatory functions *f* cannot be constant functions (i.e., cannot yield the same output regardless of the state of the regulators). In models describing signal transduction networks the external signals are represented with source nodes whose regulatory functions depend only on their own state, usually sustaining this state: σ_*i*_(*t* + τ_*i*_) = σ_*i*_(*t*).

Here we focus on discrete time Boolean network models, where node states are binary, 1(ON) or 0(OFF), and the regulatory function is specified by a truth table or using the Boolean operators AND, OR, NOT (Kauffman, 1969; Glass and Kauffman, 1973). This is motivated by the fact that biological species are frequently observed to demonstrate switch-like behaviors and have highly nonlinear regulations; thus the node state 1 means the molecular species is above a threshold concentration or activity and thus it is able to regulate its targets, and the node state 0 means it is below a threshold concentration or activity and is thus ineffective (Bornholdt, 2008; Wang et al., 2012). Depending on the updating scheme, the time trajectory of the system is simulated deterministically or stochastically. A simple deterministic updating scheme is synchronous updating, where τ_*i*_ = 1 for every node (Wang et al., 2012). In this scheme, the system will deterministically evolve from a specific initial state into an attractor, which can be a steady state (fixed point) or a limit cycle, which consists of several states that repeat regularly. Steady states can be interpreted as cell types; limit cycles may correspond to a cell cycle or circadian rhythms. In general asynchronous updating, a commonly used stochastic updating scheme, a random node is selected to be updated at each time step (Glass, 1975). This type of update is motivated by the fact that different biological processes have various timescales, and often the timescales of specific processes are not known (Papin et al., 2005). While limit cycles depend on the specific chosen updating regime, fixed points (steady states) do not depend on the updating scheme (Klemm and Bornholdt, 2005). Stochastic update may lead to attractors that involve irregular repetition of a set of states, called complex attractors.

### 2.2. The expanded network and its use in identifying the attractor repertoire of a boolean network

**Figure 1:**
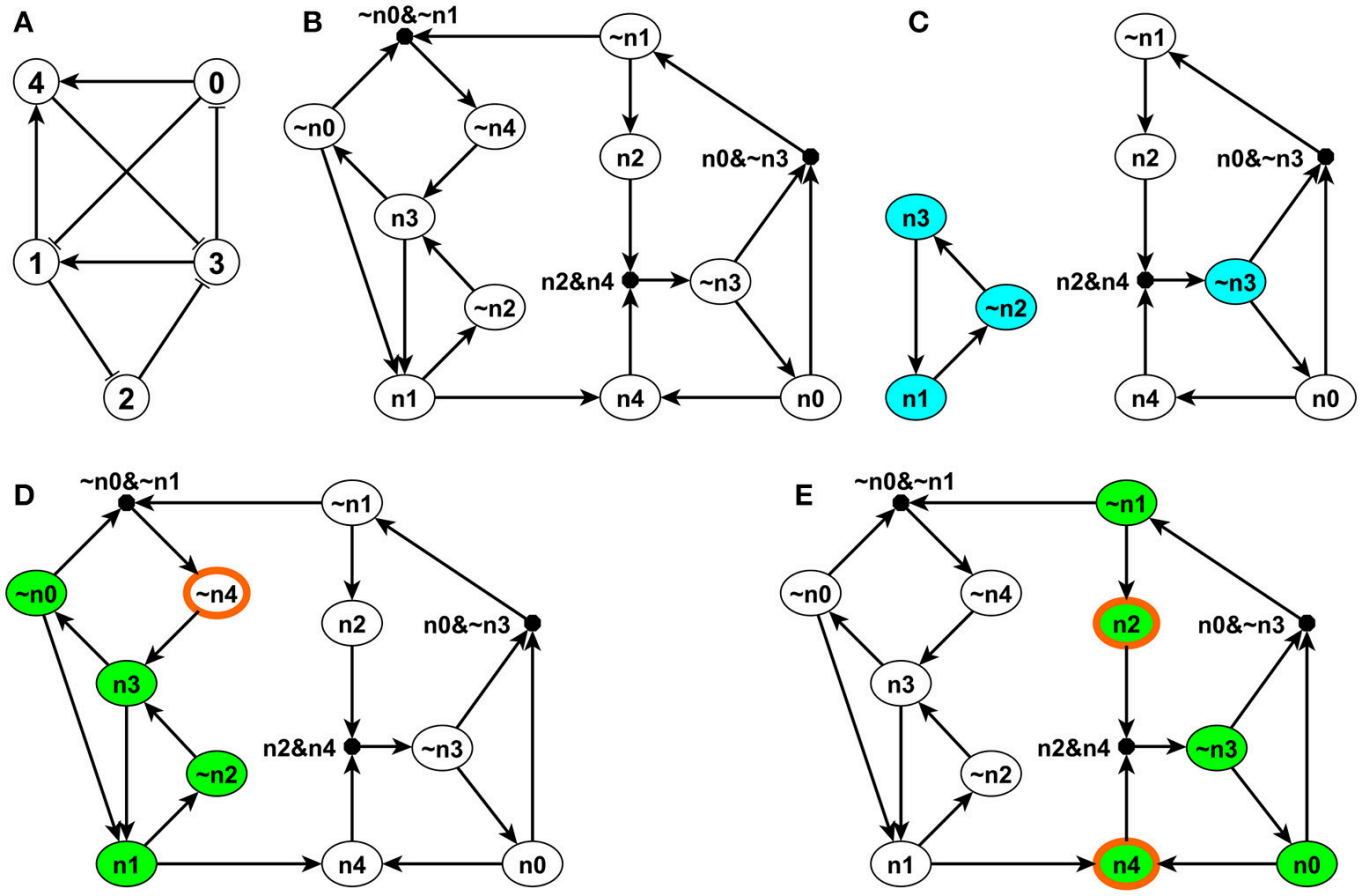
Illustration of the expanded network, stable motifs and logic domain of influence on a simple example. The network is shown in panel **(A)**. Each edge with an arrow represents activation and each edge with a flat bar represents inhibition. The Boolean regulatory functions are specified as follows: *f*_0_ = NOT σ_3_, *f*_1_ = (NOT σ_0_) OR σ_3_, *f*_2_ = NOT σ_1_, *f*_3_ = (NOT σ_2_) OR (NOT σ_4_), *f*_4_ = σ_0_ OR σ_1_. Panel **(B)** shows the expanded network of this example. Each node i in panel **(A)** has a correspondent *n*_*i*_ and its complementary node ~*n*_*i*_ in panel **(B)**. (Note that *n*_*i*_ is labeled as *ni* in panel **(B)** to be more visible). A composite node is drawn as a filled black circle and & represents the AND logic operator. Panel **(C)** indicates the stable motifs; each blue node is a single-node core of the corresponding stable motif. Panels **(D,E)** show the LDOI of {~*n*_4_} and {*n*_2_, *n*_4_}, respectively, overlaid over the expanded network. Nodes with thick orange boundary are the sustained interventions and the green nodes are their LDOI.

The possible combinatorial effect of multiple incoming regulators of a node is important, however, it is not explicitly represented by a regular interaction network. This motivated researchers to develop a concept called the expanded network, which integrates the original network with the regulatory rules of each node (Albert and Othmer, 2003; Wang and Albert, 2011). We illustrate the expanded network with the example in Figure 1, which consists of five nodes, node 0, 1, 2, 3, and 4 with the regulatory functions *f*_0_ = NOT σ_3_, *f*_1_ = (NOT σ_0_) OR σ_3_, *f*_2_ = NOT σ_1_, *f*_3_ = (NOT σ_2_) OR (NOT σ_4_), *f*_4_ = σ_0_ OR σ_1_. First, we denote each original node *i* by *n*_*i*_ in the expanded network, and we introduce a complementary node for each original node in the system to represent the negation (deactivation) of the original node, denoted by ~*n*_*i*_ (Wang and Albert, 2011). As the NOT function is a unary operator, all the NOT functions are replaced by the negated state of the respective node (i.e., its complementary node) in each Boolean regulatory function. Edges are introduced in the expanded network to represent the thus-transformed regulatory functions so that every edge represents a positive regulatory relationship in the expanded network. For example, *f*_0_ = NOT σ_3_ implies the rule for the original node *n*_0_ as *f*_*n*_0__ = NOT *n*_3_ = ~*n*_3_, and thus a corresponding edge is drawn from ~*n*_3_ to *n*_0_ in the expanded network. The Boolean regulatory function for the complementary (negated) node is the logical negation of the regulatory function of the original node. In this example, *f*_~*n*_0__ = NOT (NOT *n*_3_) = *n*_3_ and thus a corresponding edge is drawn from *n*_3_ to ~*n*_0_ in the expanded network.

Second, to differentiate OR rules from AND rules when multiple edges point toward the same target node, we introduce a composite node for each set of edges that are linked by an AND function (Wang and Albert, 2011). In order to uniquely determine the edges of the expanded network, the regulatory functions need to be specified in disjunctive normal form, that is, a disjunction of conjunctive clauses (in other words, grouped AND clauses separated by OR clauses). For example, (*A* AND *B*) OR (*A* AND *C*) is in a disjunctive normal form, while its equivalent form *A* AND (*B* OR *C*) is not. The desired disjunctive normal form can be formed by a disjunction of all conditions that give output 1 in the Boolean table and then simplified to the disjunction of prime implicants (Blake canonical form) by the Quine-McCluskey algorithm (McCluskey, 1956). Now we add a composite node for each AND clause in the Boolean regulatory function, denoted by a filled black circle in Figure 1B. We add edges from the non-composite nodes of the expanded network that form this clause to this composite node. For example, the composite node ~*n*_0_&~*n*_1_ in the left upper part of Figure 1B represents the expression (NOT *n*_0_) AND (NOT *n*_1_). The expanded network has edges from ~*n*_0_ to the composite node and from ~*n*_1_ to the composite node. This composite node expresses the regulatory function of the complementary node ~*n*_4_, namely, *f*_~*n*_4__ = NOT *f*_*n*_4__ = NOT *n*_0_ AND NOT *n*_1_ = ~*n*_0_ AND ~*n*_1_. To reflect this, the expanded network contains an edge from this composite node to ~*n*_4_. Now the benefit of introducing complementary and composite nodes is evident: one can read all the regulatory functions from the topology of the expanded network. The NOT rule is indicated by a complementary node, the AND rule is indicated by a composite node with multiple regulators, while all the other edges represent independent activation (parts of an OR function). Moreover, the expanded network also incorporates the negations of the regulatory functions. Thus, for each node *i*, the expanded network reflects the condition that needs to be satisfied in order for σ_*i*_ = 1 (in the incoming edges of the node *n*_*i*_) and the condition that needs to be satisfied in order for σ_*i*_ = 0 (in the incoming edges of the node ~*n*_*i*_).

As the expanded network encapsulates the regulatory logic that determines the network dynamics, it can serve as a basis for attractor analysis. One approach is through analyzing the stable motifs of the expanded network (Zañudo and Albert, 2013). A stable motif is defined as the smallest strongly connected component (SCC) satisfying the following two properties: (1) The SCC cannot contain both a node and its complementary node and (2) If the SCC contains a composite node, it must also contain all of its input nodes (Zañudo and Albert, 2013). The first requirement guarantees that the SCC does not contain any conflict in node states and the second requirement guarantees that all the conditional dependence is satisfied and the SCC is self-sufficient in maintaining each node state inside the stable motif. Thus the stable motif represents a group of nodes that can sustain their states irrespective of outside nodes' states. The corresponding node states implied by the stable motif can be directly read out: an original node represents the ON (1) state and a complementary node represents the OFF (0) state (Zañudo and Albert, 2013). For example, in the left part of Figure 1C, node *n*_1_, ~*n*_2_, and *n*_3_ form a stable motif, representing that node 1 and node 3 are ON and node 2 is OFF. There is a strong correspondence between stable motifs and the attractors of the system. Specifically, there is a one-to-one correspondence between a sequence of stable motifs and a fixed point or a partial fixed point (a part of a complex attractor). A partial fixed point is defined as a true subset of all the nodes whose respective state remains unchanged after being updated regardless of the states of the nodes excluded from this subset (Zañudo and Albert, 2013).

### 2.3. The domain of influence of a sustained node state

We define the DOI of an intervention that maintains a sustained node state as all the node states that will be stabilized (i.e., attain a stationary value) in the long term under the influence of this intervention for all initial conditions in any updating regime. Mathematically, $D\left(\sigma_{i}={\tilde{\sigma }}_{i}\right)=\left\{\sigma_{j}={\tilde{\sigma }}_{j}:\sigma_{j}\left(t\right)={\tilde{\sigma }}_{j}\;as\;t\to \infty \;for\;any\;\left(\sigma_{1}\left(t=0\right),\ldots ,\sigma_{k}\left(t=0\right)\right)\;when\;\sigma_{i}\left(t\right)={\tilde{\sigma }}_{i}\;for\;any\;t>0\right\}$, where $\sigma_{i}\left(t\right)={\tilde{\sigma }}_{i}$ is the intervention, ${\tilde{\sigma }}_{i}=0$ represents knockout or suppression and ${\tilde{\sigma }}_{i}=1$ represents sustained activation, ${\tilde{\sigma }}_{j}$ represents a node state fixed by the intervention, and (σ_1_(*t* = 0), …, σ_*k*_(*t* = 0)) represents the initial condition of all the nodes of the system. We do not include the intervention node state $\sigma_{i}={\tilde{\sigma }}_{i}$ in its own DOI, unless the node is sufficient to maintain the corresponding node state in the long term even in the absence of a sustained intervention. Notice that there is one-to-one correspondence between a node state $\sigma_{i}={\tilde{\sigma }}_{i}$ and a non-composite node *n*^*ex*^ in the expanded network : σ_*i*_ = 1 corresponds to a normal node *n*_*i*_ in the expanded network and σ_*i*_ = 0 corresponds to a negation node ~*n*_*i*_. Thus we use the two notations interchangeably, that is, $\sigma_{j}=1\in D\left(\sigma_{i}=1\right)$ is equivalent to $n_{j}\in D\left(n_{i}\right)$ and $\sigma_{j}=0\in D\left(\sigma_{i}=0\right)$ is equivalent to ~*n_j_* ∈ D(~*n*_*i*_).

The DOI of a node is difficult to calculate because it entails determining the common part of all attractors of a dynamical system to identify the nodes whose states stabilize due to the considered intervention. As an alternative to this computationally hard problem, we define a related concept called the LDOI of an intervention that maintains a sustained node state. The LDOI consists of all the node states that, for any initial condition, are stabilized by the first update of the corresponding node in an updating regime that preserves the level order (breadth first search order) of the expanded network. An updating regime preserves the level order if all the nodes in the *n*th level are updated at least once before updating any node in the (*n* + 1)th level (see details in Supplementary Material 2.1). We denote the LDOI of a node state σ_*i*_ as $LD\left(\sigma_{i}={\tilde{\sigma }}_{i}\right)$. We define the LDOI of an empty set to be an empty set, LD(Ø) = Ø. This is consistent with the definition as an updating order preserving the level order starting from a null set can start from any node, and a node will not achieve a stationary state upon its very first update for all initial conditions unless its regulatory function is a constant. Source nodes remain in their initial state, which nevertheless will be different for different initial conditions.

### 2.4. Determining the logical domain of influence of a sustained node state

We propose to find the LDOI of a node state by doing a modified breadth first search (BFS) on the expanded network (see the pseudocode in Supplementary Material section 1.1). In order to find the LDOI of $\sigma_{i}={\tilde{\sigma }}_{i}$, we start the search from the corresponding node *n*_*i*_ on the expanded network if σ_*i*_ = 1 or we start the search from the complementary node ~*n*_*i*_ if σ_*i*_ = 0. If we meet another non-composite node, we add this node to the LDOI; if we meet a composite node, we add this composite node only if all of its parent nodes (i.e., regulators) are already part of the LDOI. This is due to the fact that any edge from a node to a non-composite node represents a sufficient relationship and any edge from a node to a composite node represents a necessary relationship. We keep searching on the expanded network until no new nodes can be added to the LDOI. For example, in Figure 1B, one can readily see that LD(σ_1_ = 1) ≡ LD(*n*_1_) = {*n*_4_,~*n*_2_, *n*_3_, *n*_1_, ~*n*_0_} following the described search procedure. The first difference from a normal BFS to find a connected component starting from a node is that we put an extra rule for including a composite node. Another subtle difference is that we do not include the starting point unless we visit this starting point again in our search process.

During the search process, there is a possibility that we meet the negation of the starting point. This reflects the possibility that a node state can indirectly lead to the opposite state through a negative feedback loop. This outcome represents a conflict with the original intervention. We do not add this node to the LDOI because we assume that the intervention can sustain the original node state, thus the opposite state is not reachable. This truncation of the LDOI to avoid including the negation of the starting node state ensures that the LDOI will not contain a node which is the negation of an already visited node. Mathematically, if a non-composite node $n_{i}^{ex}\in LD\left(n_{j}^{ex}\right)$, then $n_{j}^{ex}$ is sufficient to activate $n_{i}^{ex}$, i.e., the long-term logical rule for $n_{i}^{ex}$ can be expressed in the form $n_{i}^{ex}=n_{j}^{ex}\text{OR}\cdots \;$; this implies ~$n_{i}^{ex}=$~$n_{j}^{ex}\text{AND}\cdots \;$, i.e., ~$n_{j}^{ex}$ is necessary to activate ~$n_{i}^{ex}$. Thus any conflict between $n_{i}^{ex}$ and ~$n_{i}^{ex}$ will occur after the conflict between $n_{j}^{ex}$ and ~$n_{j}^{ex}$ during the search process. This truncation of the LDOI is the third difference compared with a normal BFS.

For example, in the network of Figure 1D, the LDOI of the complementary node ~*n*_4_ includes nodes *n*_3_, ~*n*_0_, *n*_1_, ~*n*_2_ following the search procedure. From *n*_1_ one can also reach node *n*_4_, which is the negation of the considered intervention. Thus we stopped this branch of searching based on our truncation rule. Since there are no more nodes that can be added, we conclude that LD(~*n*_4_) = {*n*_3_, ~*n*_0_, *n*_1_, ~*n*_2_}.

Our LDOI search procedure is equivalent to doing a simulation on the expanded network. If we update the system corresponding to the BFS order of the expanded network starting from the intervention node (i.e., we update node *i* if we visited $n_{i}^{ex}$ on the expanded network), all the updated nodes are guaranteed to stabilize in the corresponding visited state on the expanded network, i.e., as in the LDOI of that node. In the example of Figure 1, as discussed above, LD(*n*_1_) = {*n*_4_,~*n*_2_, *n*_3_, *n*_1_, ~*n*_0_}. If we update the nodes in the order 4, 2, 3, 1, 0, each node will stabilize in the state as in $LD\left(n_{1}\right)$. We note that this does not put a restriction on the updating regime: if we update the system in an arbitrary order, each node in the LDOI of the given sustained intervention will attain a stationary state in the first update after all of its regulators included in the LDOI have been updated once. For example, if we fixed the node 1 to be ON and we perform rounds of update of the nodes in the order 0, 1, 2, 3, 4, nodes 2, 3, and 4 will be stabilized in the first round of updating, while nodes 0 and 1 will be stabilized in the second round.

**Figure 2:**
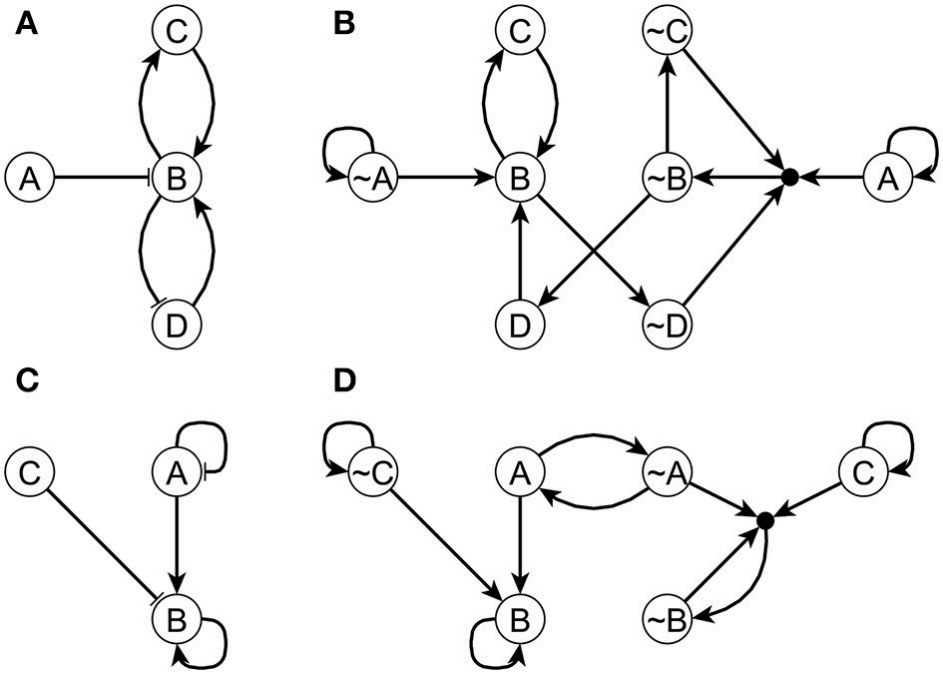
Two example networks **(A,C)** and their respective expanded networks **(B,D)** that illustrate the difference between DOI and LDOI. In both networks, an edge with an arrowhead represents activation while an edge with flat bar represents inhibition. Implicit positive self-loops stemming from the assumed sustained states of source nodes are not shown in panels **(A,C)**. In panel **(A)** the regulatory functions are *f*_*A*_ = *A*, *f*_*B*_ = (NOT *A*) OR *C* OR *D*, *f*_*C*_ = *B, f*_*D*_ = NOT *B* . When A = 1 the system has a fixed point σ_*B*_ = σ_*C*_ = 1, σ_*D*_ = 0. In panel **(C)** the regulatory functions are *f*_*A*_ = NOT *A*, *f*_*B*_ = *A* OR *B* OR NOT *C, f*_*C*_ = *C*. When σ_*C*_ = 1 the system has a complex attractor in which A oscillates and σ_*B*_ = 1.

The difference between the LDOI and DOI is that LDOI requires the nodes to be stabilized when being updated for the first time, while DOI just requires the nodes to be stabilized in finite time. Thus one can see that the LDOI of a node will be a subset of the DOI of a node. In many cases the two concepts give the same result. Two exceptions are illustrated in Figure 2. In both cases the DOI of an intervention contains more nodes than the LDOI of this intervention. This is because certain nodes may stabilize not because of the influence of the intervention but because of the collective effect of two inconsistent feedback loops or because of a stable motif stabilized by an oscillation. In the network of Figure 2A, the three regulators of node B are independent and the network includes both a positive and a negative feedback loop. To analyze the LDOI of *A* = 1, taking the feedback effect of C and D on B into consideration, the regulatory function of B is simplified into σ_*B*_(*t* + τ_*B*_) = σ_*B*_(*t* − τ_*C*_) OR NOT (σ_*B*_(*t* − τ_*D*_)), where τ_*i*_ is the discrete time delay for node *i*, as introduced in section 2.1. This regulatory function admits a constant solution σ_*B*_ = 1 regardless of the values of the time delays (Saadatpour et al., 2010; Azuma et al., 2014). It may additionally admit an oscillatory solution for strict relationships among the time delays. In the cases where there is no oscillatory solution, for example in the cases where only one node can change state at a time, D(*A*) = {*B*, *C*,~*D*}, as the stabilization of B leads to the stabilization of C and D as well. However, LD(A)= as the activation of the composite node requires nodes *A*, ~*C*, ~*D* on the expanded network shown in Figure 2B and thus we cannot add the composite node to the LDOI of node A. In the example shown in Figure 2C, the two regulators are independent for node B, D(C)={B} as the negative feedback loop of node A will make A oscillate, but B will stabilize into the ON state after the first time that A visits the ON state and activates B, while LD(C)= for the same reason as in the last example.

### 2.5. Properties of the logical domain of influence of a sustained node state

In order to further illustrate the concept of LDOI, we discuss a few of its properties and its relationship with established concepts in Boolean dynamics. The LDOI of a node state is mathematically equivalent to the three-valued logical steady state that results when this node state is fixed (Klamt et al., 2006; Samaga et al., 2010). Here the three values are 0, 1, and unknown (for nodes who do not attain a stationary state solely due to the original node's fixed state). The LDOI of a node state is also equivalent to the set of nodes, and corresponding states, that are identified using network reduction techniques [i.e., by iteratively substituting the fixed node state(s) into the regulatory function(s) of target nodes] (Bilke and Sjunnesson, 2001; Naldi et al., 2012; Saadatpour et al., 2013). Previous analysis (Samaga et al., 2010; Saadatpour et al., 2013) identified that if node *i* has a single outgoing edge, and is a sufficient regulator of its sole target node, *j*, the LDOI of the ON state of *i* contains the LDOI of the ON state of *j*. Here we study in general the possible inclusion relationship between the logic domains of influence of two node states $\sigma_{i}={\tilde{\sigma }}_{i}$ and $\sigma_{j}={\tilde{\sigma }}_{j}$ in the case when $\sigma_{j}={\tilde{\sigma }}_{j}\in LD\left(\sigma_{i}={\tilde{\sigma }}_{i}\right)$ or $n_{j}^{ex}\in LD\left(n_{i}^{ex}\right)$ in the expanded network notation, where $n_{i}^{ex}$ and $n_{j}^{ex}$ represent any non-composite node in the expanded network. In a directed graph, if node *n*_*j*_ is a reachable from node *n*_*i*_, all descendants of *n*_*j*_ will also be reachable from *n*_*i*_; indeed one can easily prove this by contradiction. However, due to the special properties of the expanded network and the truncation of the LDOI, this inclusion relationship $LD\left(n_{j}^{ex}\right)\subseteq LD\left(n_{i}^{ex}\right)$ is not generally true for the expanded network. It is possible that $n_{j}^{ex}\in LD\left(n_{i}^{ex}\right)$, however, ~$n_{i}^{ex}\in LD\left(n_{j}^{ex}\right)$. In this case, by definition of the LDOI, we won't allow the negation of a node state to be part of the LDOI of a node state. For example, *n*_1_ ∈ LD(~*n*_4_), however, $n_{4}\in LD\left(n_{1}\right)$. Thus LD(*n*_1_) ⊄ LD(~*n*_4_).

If we add an additional restriction on the two nodes, this inclusion relationship will hold the same way as for descendants in a directed graph. To be specific, the *first key property of the LDOI* is, if the node state $\sigma_{i}={\tilde{\sigma }}_{i}$ and $\sigma_{j}={\tilde{\sigma }}_{j}$, corresponding to the two non-composite node $n_{i}^{ex}$ and $n_{j}^{ex}$ on the expanded network, are both included in the same (partial) fixed point and $n_{j}^{ex}\in LD\left(n_{i}^{ex}\right)$, the LDOI of $n_{j}^{ex}$ will be a subset of the LDOI of $n_{i}^{ex}$, i.e., $LD\left(n_{j}^{ex}\right)\subseteq LD\left(n_{i}^{ex}\right)$. (Recall that a partial fixed point is a subset of nodes whose respective state remains unchanged after being updated regardless of the states of the nodes excluded from this subset.) The reason why the inclusion relationship holds is that node states in a (partial) fixed point stabilize in the long term, thus they will not lead to a situation with opposing behavior $n_{j}^{ex}\in LD\left(n_{i}^{ex}\right)$ and ~$n_{i}^{ex}\in LD\left(n_{j}^{ex}\right)$. This restriction can be weakened to only require that node state $n_{i}^{ex}$ is in a (partial) fixed point. The reason is that if $n_{j}^{ex}\in LD\left(n_{i}^{ex}\right)$ and $n_{i}^{ex}$ is in a (partial) fixed point, then $n_{j}^{ex}$ must also be in the same (partial) fixed point, or be a node whose state stabilizes due to the nodes in the partial fixed point. Also, as one or more stable motifs are part of a (partial) fixed point, the conclusion will be true if one replaces “(partial) fixed point” by “stable motif” in the above statement. In the example of Figure 1, as nodes *n*_1_, ~*n*_2_ and *n*_3_ form a stable motif and its corresponding (partial) fixed point is (σ_1_, σ_2_, σ_3_) = (1, 0, 1) as shown in Figure 1C, which also lead to the stabilization of the remaining two nodes as σ_0_ = 0 and σ_4_ = 1, thus $n_{3}\in LD\left(n_{1}\right)$ implies that $LD\left(n_{3}\right)\subseteq LD\left(n_{1}\right)$. In fact, LD(*n*_3_) = LD(*n*_1_) = {*n*_4_,~*n*_2_, *n*_3_, *n*_1_, ~*n*_0_}. Also $n_{4}\in LD\left(n_{1}\right)$ implies that $LD\left(n_{4}\right)\subseteq LD\left(n_{1}\right)$. Note that only *n*_1_ is part of the stable motif or partial fixed point in the latter example, *n*_4_ is not.

As stable motifs represent generalized positive feedback loops of the Boolean network (Zañudo and Albert, 2013), we explore the relationship between stable motifs and the LDOI of a node state. The *second key property of LDOI* is, if the LDOI of a node state contains this node state itself, the LDOI contains a stable motif. As the LDOI of a node state only contains the node state itself if we meet this node during the search process on the expanded network, this indicates the existence of a positive feedback loop, which is the intuition why this proposition holds. (A sketch of proof from the dynamical standpoint is included in Supplementary Material section 2.2). For example, $n_{1}\in LD\left(n_{1}\right)$ implies that there exists a stable motif contained in $LD\left(n_{1}\right)$, indeed, *SM*_1_ = {*n*_1_, ~*n*_2_,*n*_3_} ⊆ LD(*n*_1_).

### 2.6. The domain of influence of a node state set

Now we generalize the concept of DOI of a single node state to DOI of a node state set (i.e., a set of nodes, each in a sustained state). We define the DOI of a node state set as all the node states that can be stabilized in the long term by the given set of node states under all initial conditions in any updating regime. Mathematically, $D\left(\left\{\sigma_{i}={\tilde{\sigma }}_{i}\right\}\right)=\left\{\left\{\sigma_{j}={\tilde{\sigma }}_{j}\right\}:\sigma_{j}\left(t\right)={\tilde{\sigma }}_{j}ast\to \infty forany\left(\sigma_{1}\left(t=0\right),\ldots ,\sigma_{k}\left(t=0\right)\right)when\sigma_{i}\left(t\right)={\tilde{\sigma }}_{i}foranyt>0\right\}$, where $\left\{\sigma_{i}\left(t\right)={\tilde{\sigma }}_{i}\right\}$ represents the intervention consisting of a specific set of node states. Note that the following two notations are equivalent: $D\left(\left\{\sigma_{i}={\tilde{\sigma }}_{i}\right\}\right)\equiv D\left(\left\{n_{i}^{ex}\right\}\right)$. Similarly, we define the LDOI of a node state set, $LD\left(\left\{\sigma_{i}={\tilde{\sigma }}_{i}\right\}\right)$, as all the nodes that can be stabilized by the first update in any BFS order-preserving (on the expanded network) update order starting from this given set of node states under all initial conditions. As in the single node state case, the LDOI of a node state set will be a subset of the DOI of the same node state set.

The LDOI of a node state set can be determined by a modified BFS on the expanded network, now using multiple starting points. This does not add complexity to the iterative implementation of BFS: we just need to initialize the queue with the set of given node states. Similar to the case of finding the LDOI of a single node state, we need to deal with the conflicts that may occur during the search process. To be precise, conflict means that during the search we visit a node state that is the negation of a node state included in the intervention. Two types of conflict can arise. First, a node state in the given set may be impacted by negative feedback and have a LDOI that was truncated to avoid containing its own negation. Second, the LDOI of two node states $n_{i}^{ex}$ and $n_{j}^{ex}$ may have the property ~$n_{i}^{ex}\in LD\left(n_{j}^{ex}\right)$ or ~$n_{j}^{ex}\in LD\left(n_{i}^{ex}\right)$, or both. In other words, node *i* may regulate node *j* (or *vice versa*) in a way that is incompatible with the intervention (e.g., a node whose sustained activity is part of the intervention may negatively regulate another node whose sustained activity is part of the intervention). We call intervention sets that have either type of conflict incompatible sets; we refer to the rest of the intervention sets as compatible sets. Similarly to the truncation we did to find the LDOI of a single node state, we do not include any node state that is the negation of any node state given in the intervention set and we stop searching that branch. We note that this truncation strategy avoids any following conflict. For example, if $n_{C}\in LD\left(n_{A}\right)$ and ~$n_{C}\in LD\left(n_{B}\right)$, then one may expect that the LDOI of the set {*n*_*A*_, *n*_*B*_} will have a conflict between *n*_*C*_ and ~*n*_*C*_. However, $n_{C}\in LD\left(n_{A}\right)$ implies that ~*n*_*C*_ requires ~*n*_*A*_, this means that meeting the conflict between *n*_*C*_ and ~*n*_*C*_, must be after meeting the conflict between *n*_*A*_ and ~*n*_*A*_, which is avoided by our truncation strategy.

For a compatible set $\left\{n_{i}^{ex}\right\}\equiv \cup_{i}n_{i}^{ex}$, it is guaranteed that $\cup_{i}LD\left(n_{i}^{ex}\right)\subseteq LD\left(\cup_{i}n_{i}^{ex}\right)$. For example, as shown in Figure 1E, the node set {*n*_2_, *n*_4_} is a compatible node set as $LD\left(n_{2}\right)=$, $LD\left(n_{4}\right)=$ and LD({*n*_2_,*n*_4_}) = {~*n*_3_, *n*_0_, *n*_4_, ~*n*_1_, *n*_2_}. Note $LD\left(n_{2}\right)\cup LD\left(n_{4}\right)\subseteq LD\left(\left\{n_{2},n_{4}\right\}\right)$. However, for an incompatible set, we just know that the situation $\cup_{i}LD\left(n_{i}^{ex}\right)⊊LD\left(\cup_{i}n_{i}^{ex}\right)$ cannot happen and all the remaining situations are possible. In the network of Figure 1, node set {*n*_2_, ~*n*_4_} is an incompatible node set as $LD\left(n_{2}\right)=$, LD(~*n*_4_) = {*n*_3_, ~*n*_0_, *n*_1_, ~*n*_2_}, and LD({*n*_2_,~*n*_4_}) = {*n*_3_, ~*n*_0_, *n*_1_}. Note that neither *n*_4_ nor ~*n*_2_ are included in LD({*n*_2_,~*n*_4_}) due to the truncation rule and LD({*n*_2_,~*n*_4_}) ⊊ LD(*n*_2_) ∪ LD(~*n*_4_). Node set {~*n*_1_, *n*_3_} is another incompatible set as LD(~*n*_1_) = {*n*_2_}, LD(*n*_3_) = {~*n*_0_, *n*_1_, ~*n*_2_, *n*_4_, *n*_3_} and LD({~*n*_1_, *n*_3_}) = {*n*_2_, ~*n*_0_, ~*n*_4_, *n*_3_}. Note that LD({~*n*_1_,*n*_3_}) ⊄ LD(~*n*_1_) ∪ LD(*n*_3_), and LD(~*n*_1_)∪ LD(*n*_3_) ⊄ LD({~*n*_1_, *n*_3_}).

The properties of the LDOI of a single node can also be generalized to the LDOI of a given node set. For the first key property, let $S_{j}=\left\{\sigma_{j}={\tilde{\sigma }}_{j}\right\}$ and $S_{i}=\left\{\sigma_{i}={\tilde{\sigma }}_{i}\right\}$ be two sets of node states, if *S*_*i*_ is a subset of any (partial) fixed point and $S_{j}\subseteq LD\left(S_{i}\right)$, then $LD\left(S_{j}\right)\subseteq LD\left(S_{i}\right)$. The intuition is similar, the requirement restricting us to consider those nodes which can be stabilized in the long term, that is, we rule out the possibility of *S*_*i*_ being an incompatible node set. For example in Figure 1 consider *S*_*i*_ = {~*n*_3_} and *S*_*j*_ = {*n*_2_, *n*_4_}. As ~*n*_3_ is part of the stable motif *SM*_2_ = {*n*_0_, ~*n*_1_, *n*_2_, ~*n*_3_, *n*_4_}, corresponding to the fixed point (σ_0_, σ_1_, σ_2_, σ_3_, σ_4_) = (1, 0, 1, 0, 1), $S_{j}\subset LD\left(S_{i}\right)$ implies $LD\left(S_{j}\right)\subseteq LD\left(S_{i}\right)$. In fact, $LD\left(S_{j}\right)=LD\left(S_{i}\right)$.

The second key property also generalizes: if the LDOI of a given node state set contains the set itself, then the LDOI of the set contains at least one stable motif. The intuition and proof is similar to the case of a single node state. Taking the same example, consider *S*_*i*_ = {~*n*_3_} and *S*_*j*_ = {*n*_2_, *n*_4_}, note that both $S_{i}\subset LD\left(S_{i}\right)$ and $S_{j}\subset LD\left(S_{j}\right)$, this implies that both $LD\left(S_{i}\right)$ and $LD\left(S_{j}\right)$ contain a stable motif, which is *SM*_2_ in this case.

Following these examples, we define the core of a stable motif to be a minimal subset of the stable motif whose LDOI contains the stable motif. Here by minimal we mean that no true subset of the core of the stable motif will contain the entire stable motif. The core of a stable motif can be a single node or more than one node. For example, as shown in Figure 1C ~*n*_3_ is a single-node core of the stable motif *SM*_2_ = {*n*_0_, ~*n*_1_, *n*_2_, ~*n*_3_, *n*_4_}. {*n*_2_, *n*_4_} is another core of the same stable motif as $SM_{2}⊄LD\left(n_{2}\right)$, $SM_{2}⊄LD\left(n_{4}\right)$, and $SM_{2}\subseteq LD\left(\left\{n_{2},n_{4}\right\}\right)$.

We also define a driver node (set) of the stable motif to be a node (set) whose DOI contains the entire stable motif. The driver node (set) can be inside the stable motif, in which case it is the core of the stable motif; it can also be an upstream node that is sufficient to activate (the core of) the stable motif. We note that stabilization of a stable motif does not require the sustained state of a driver node, that is, oscillations can also lead to the stabilization of a stable motif. An example of this behavior was shown in Figure 2B: node B, which constitutes a self-sustaining stable motif, can stabilize by a single instance of A = 1, regardless of the fact that the negative self-regulation of A makes it oscillate.

### 2.7. Target control algorithm

Now that we have equipped ourselves with the tool of LDOI to find the long term effect of a sustained intervention, we can formulate the target control problem as the identification of a node set S^*^ whose LDOI contains the target node state set, i.e., $LD\left(S^{*}\right)⊇Target$. This problem can be framed as a planning search problem (Russell and Norvig, 2003). We start with a null set whose LDOI is also null. We repeatedly add a new node to the set until the LDOI of this set contains the target node state set. We use LDOI instead of DOI for this purpose because identification of the DOI is a computationally more difficult problem. Our current solution using LDOI sets a tight upper bound for the optimal solution for the target control problem as $D\left(S^{*}\right)⊇LD\left(S^{*}\right)⊇Target$.

Previous work in the target control of Boolean models has focused on full enumeration of the solutions for the target control problem (Klamt et al., 2006; Samaga et al., 2010), which can be used to identify the solutions that involve combinations of a small number of nodes but is not generally viable because of combinatorial explosion. In our work, we use a complementary approach to avoid a full state space search in this combinatorial search problem. We apply a random heuristic algorithm called the greedy randomized adaptive search procedure (GRASP) (Pardalos et al., 1998; Festa et al., 2001). The pseudocode is described in Algorithm (Tables 1, 2). The algorithm consists of two main phases. The first phase is the construction of a greedy randomized solution and the second phase is a local search to remove any redundancy of the solution.

**Algorithm 1:** GRASP algorithm for Target Control Problem

1: **procedure** GRASP(*G*_*expanded*,*Target*,*max*_*itr*)
2: *solutions* ← *List*()
3: **for** *index* ← 1, *max*_*itr* **do**
4: *solution* ← ConstructGreedyRandomizedSolution (*G*_*expanded, Target*)
5: *solution* ← LocalSearch(*G*_*expanded, Target, solution*)
6: **if** *solution* **then**
7: *Solutions*.*append*(*solution*)
8: **end if**
9: **end for**
10: **return** *solutions*
11: **end procedure**

**Algorithm 2:** Algorithm for constructing a greedy randomized solution

1: **procedure** ConstructGreedyRandomizedSolution (*G*_*expanded, Target*)
2: *solution* ← *Set*()
3: α ← *random*(0, 1)
4: *candidates* ← Construct_Initial_Candidates(*G*_*expanded, Target*)
5: *G*(*v*) ← Construct_Greedy_Functions(*G*_*expanded, candidates*)
6: **while** *candidates* **do**
7: *RCL* ← MakeRCL(*candidates, G*(*v*), α)
8: *s* ← Select_Candidate(*RCL*)
9: *solution* ← *solution*∪{*s*}
10: **if** *Target* ⊂ *LDOI*(*solution*) **then**
11: **return** *solution*
12: **end if**
13: Update_Candidates(*candidates*)
14: **end while**
15: **return** *Set*()
16: **end procedure**

**Table 1:** Mean number of solutions found for each target set and random network pair for 50 target sets and 1,000 networks.

**Custom score index and notation**	**1 *LDOI***	**2 *Comp*_*LDOI***	**3 *Scores*_1+2**	**4 *LDOI*_*Pen*1**	**5 *LDOI*_*Pen*2**
Nested canalizing rules	12.29	31.66	12.30	31.21	40.64
Effective boolean rules	26.21	61.94	26.22	57.08	66.91

*Half of 50 target sets have size two and the other half is of size three; none of them contain source nodes. The 2nd to 6th columns correspond to different custom score (greedy function) indexes and notations, which are described in the last paragraph in section 2.7. The second and third row corresponds to the random network ensemble with nested canalizing rules and effective Boolean rules, respectively*.

**Table 2:** Summary of representative target control solutions found by our algorithm for four biological network models.

**Model**	**Target state(s)**	**Representative interventions found**	**Previous results from dynamic analysis**	
			**EMT-blocking**	**E cell inducing**
EMT network	~EMT	β-catenin_memb = 1, SNAI1 = 0	SNAI = 0	β-catenin_memb = 1, SMAD = RAS = 0, SNAI1 = GLI = 0
		β-catenin_memb = 1, SMAD = RAS = 0	SMAD = 0, RAS = 0	
	~EMT, ~MEK	β-catenin_memb = 1, SNAI1 = RAS = 0	Not studied previously	
		β-catenin_memb = 1, miR200 = RKIP = 1, RAS = 0		
Breast cancer network	Apoptosis = 2, Proliferation = 0	PI3K = 0, ESR1 = 0	PI3K = 0, ESR1 = 0	
	Apoptosis < 2, Proliferation >2 when PI3K = 0	PI3K = 0, ESR1 = 1	PI3K = 0, ESR1 = 1	
ABA induced closure network	Closure = 1 when ABA = 0	$Ca_{c}^{2+}$ = ROS = 1	ROS = 1	
	*H*_2_*O* efflux when ABA = 0	*K*^+^ efflux = 1, SLAC1 = ROS = 1	Not studied previously	
T-LGL leukemia network	Apoptosis = 1	S1P = 0, RAS = 1	S1P = 0	

*The first column indicates the relevant network model, the second lists one or two target states we considered, the third column presents the intervention set obtained by our algorithm, and the fourth column indicates the most relevant results of previous analysis of these network models. The previous analysis considered a unique initial state or a restricted family of initial states (in the case of the ABA network). The interventions found by our algorithm will be successful regardless of the initial state*.

In the first phase, we first generate an initial candidate list (line 4 in Algorithm 2). In the simplest case, the initial candidate list is all the non-composite nodes of the expanded network except the nodes in the target set and their negation, both of which are ineligible for control. One can also be more selective to adapt to the specific needs of controlling biological systems. For example, we can forbid the use of certain nodes or node states when constructing the initial candidate list, to incorporate the fact that certain chemical species are harder or even unrealistic to control. Thus these nodes/chemical species will never appear in the final solution since they are not in the initial candidate list.

Then, we begin the procedure of iteratively adding nodes to the trial solution set (which is initially empty) and evaluating whether the LDOI of the trial solution set covers the target set. We form a restricted candidate list (RCL, line 7 in Algorithm 2) based on a greedy measure *G*(*v*) defined for each candidate node *v* in the candidate list (line 5 in Algorithm 2). A greedy function is a heuristic score to estimate whether this node should be included in the solution. We evaluated five choices of *G*(*v*), as described at the end of this section and in section 3.1. We determine the minimum score *G*_*min*_ = *min*_*v*∈*V*_*G*(*v*) and maximum score *G*_*max*_ = *max*_*v*∈*V*_*G*(*v*) among the heuristic scores of all the nodes. Then we use a previously generated random number α from a uniform distribution between 0 and 1 to set a passing score for the RCL as *G*_*pass*_ = *G*_*min*_ + α · (*G*_*max*_ − *G*_*min*_). Then the RCL consist of nodes whose greedy function is no less than the passing score, i.e., *RCL* = {*v* ∈ *V*|*G*(*v*) ≥ *G*_*pass*_}. This procedure of generating RCL is summarized in Supplementary Material section 1.3.

Next we randomly pick a node from the RCL and add it to the current trial solution (line 8 and 9 in Algorithm 2). The trial solution is used as the *source* node set of the LDOI algorithm (whose pseudocode is presented in Supplementary Material section 1.1). If the target set is contained in the set of nodes returned by the LDOI algorithm, we end the first phase and start the second phase (local search procedure) with this candidate solution (line 10 and 11 in Algorithm 2). Otherwise, we update the candidate node set and start the next iteration toward adding another node from the RCL to the trial solution set. We update the candidate node set by removing the previously added node, its negation and any node in the LDOI of the current trial solution (line 13 in Algorithm 2). We do this latter exclusion because these nodes will stabilize because of the current trial solution, and it is useless to add any stabilized state to the trial solution. We repeat the whole procedure including selecting a node randomly from the candidate set as long as there are still candidate nodes (line 6 in Algorithm 2). We return an empty set if we do not find a solution (line 15 in Algorithm 2).

In the second phase (see the pseudocode in Supplementary Material section 1.2), we start with a candidate solution that covers the target set. We randomize the order of nodes in the candidate solution and then iteratively attempt to remove each node. If after removing this node the LDOI of the modified solution still covers the target set, then we replace the candidate solution with the modified solution. Thus after one iteration of traversing all the nodes, we obtain a final solution. At worst, no node is removed from the set and the final solution is the same as the candidate solution. The randomness in the removal order provides a possibility for obtaining different minimal solutions from the same candidate solution.

In this random heuristic algorithm, we introduce two aspects of randomness in the construction phase, one is the randomness of the passing score by a different α for each iteration of solution generation process (line 3 in Algorithm 1) and another is the random selection of a node each time from the RCL inside each solution generation process (line 8 in Algorithm 2). These techniques help strike a balance between the bias of a greedy function and exploring the whole node state space (Pardalos et al., 1998; Festa et al., 2001). An efficient greedy function/ heuristic score is important to guide the search procedure toward the subspace with the optimal solution. However, a universally efficient greedy function may not exist; rather, the efficiency of a greedy function may depend on the specific network structure and target set. We have implemented five choices of greedy functions *G*(*v*) for a given node state (equivalently, non-composite node of the expanded network): score 1 is the size of the LDOI of that node state (denoted as |*LDOI*|); score 2 is the size of the set of composite nodes which are nearest neighbors of the LDOI of that node state (denoted as |*Comp*_*LDOI*|); score 3 is a linear combination of the previous two measures with equal weight (denoted as *Scores*_1 + 2), and score 4 and 5 as the size of the LDOI of that node state with penalty if the LDOI contains a node that is the negation of a node in the target set (denoted as |*LDOI*|_*Pen*1 and |*LDOI*|_*Pen*2). The penalty can be implemented by multiplying this score by -1 (score 4) or by decreasing this score by the size of the largest LDOI among all node states (score 5); both of these implementations ensure that this score becomes non-positive. A python implementation of the target control algorithm is available at https://github.com/yanggangthu/BooleanDOI.

### 2.8. Computational complexity of the target control algorithm

The time complexity of calculating the LDOI of any set is bounded by *O*(*N*_*ex*_ + *E*_*ex*_), where *N*_*ex*_ is the number of nodes and *E*_*ex*_ is the number of edges of the expanded network. For each non-composite node in the network, we initially calculate its LDOI and the value of its greedy function, with time complexity *O*(*N*(*N*_*ex*_ + *E*_*ex*_)), where N is the number of nodes in the original network. We then cache these results to improve the performance of the GRASP algorithm. In the first phase of the GRASP algorithm, we run at most *N* iterations and we need to calculate the LDOI of the trial solution in each iteration, thus the time complexity is bounded by *O*(*N*(*N*_*ex*_ + *E*_*ex*_)). In the second phase, the time complexity is also bounded by *O*(*N*(*N*_*ex*_ + *E*_*ex*_)) as we need to go through each node, bounded by *O*(*N*) as a crude estimate, delete the node from the solution and check the modified solution's LDOI, which is *O*(*N*_*ex*_ + *E*_*ex*_). The Boolean regulatory functions of biological network models are often nested canalizing rules (Kauffman et al., 2003; Li et al., 2013), thus for each node with k regulators there are at most *k* newly generated composite nodes in the expanded network, as well as two corresponding non-composite nodes; each of these nodes have at most k regulators. Thus *N*_*ex*_ is bounded by $O\left(\bar{k}N\right)$, and *E*_*ex*_ is bounded by $O\left(\bar{k^{2}}N\right)$. Biological networks are sparse, with an average node in-degree $1<\bar{k}<3$ (Newman, 2010). Thus the complexity of the target control algorithm applied to biological network models is $O\left(\bar{k^{2}}N^{2}\right)\sim O\left(N^{2}\right)$ for a well-behaved degree distribution in the sparse limit and bounded by *O*(*N*^3^) for an extremely skewed degree distribution in the sparse limit. Different iterations of the solution generation process (line 3 in Algorithm 1) can be easily parallelized as each iteration is independent. The space complexity of BFS search on the expanded graph is bounded by *O*(*N*_*ex*_), and the space complexity of the entire procedure is bounded by time complexity times the storage space of LDOI of a node set, which is bounded by *O*(*N*_*ex*_).

### 2.9. Damage mitigation as target control

We can generalize the target control algorithm to solve a damage mitigation problem. Consider a Boolean network that has two steady states, one corresponding to the normal state of the system and the other corresponding to a disease state. The system is currently in the normal steady state, but damage to a node, which causes it to stabilize in the opposite state, will lead the system to the disease steady state without any intervention. Under such conditions, previous research has proposed modifying the network topology (as soon as possible, or preventatively) to block the propagation of damage (Yang et al., 2016). Here we are interested in designing a damage mitigation strategy to bring the system back to an attractor similar to the normal steady state in the sense that a subset of nodes are in the same state as their states in the normal steady state. This problem is almost the same as the target control problem except that we need to take the permanent damage into consideration. There are two ways of incorporating this. First, we treat this permanent damage as an initial condition and apply network reduction to the system. However, this risks reducing a significant fraction of the nodes in the network, including the target nodes we are interested in. Second, we can apply our GRASP algorithm as above while initializing the solution with the damaged node state(s) and forbidding the damaged node state to be removed in the local search phase in GRASP algorithm. This means that we include the damage as part of the intervention. When the LDOI of the node state set containing the damage effect covers the target set, the target nodes will stabilize in their desired states after a finite number of time steps under all initial conditions of the subspace of the damaged network. We note that we only need to do this when the damage is a permanent one; when the damage is temporary (i.e., when the node is allowed to go back to its original state), this can be treated as a different initial condition for the target control problem and we can still apply our GRASP algorithm to solve it as DOI/LDOI is robust to any initial condition by definition.

## 3. Results

### 3.1. Application to ensembles of random boolean networks

We tested the two proposed properties of the LDOI and the target control algorithm on different random Boolean network ensembles. Specifically, we generated an ensemble of 1000 Erdős Rényi random graphs (Newman, 2010) (using the gnm_random_graph() function of NetworkX; Hagberg et al., 2008), with size ranging from 15 to 50 nodes and average in-degree ranging from 1 to 2 . The Boolean regulatory functions of the random ensemble are required to be effective (irreducible) Boolean functions (Zertuche, 2009) to be consistent with the generated topology, or nested canalizing functions to simulate biological systems. (A nested canalizing Boolean function with *k* inputs can be generated by determining two sequences, the input sequence (*I*_*1*_, *I*_*2*_, ⋯, *I*_*k*_) and the output sequence (*O*_*1*_, *O*_*2*_, ⋯, *O*_*k*_), where *I*_*i*_ or *O*_*i*_ is either 0 or 1. The output *o* as a function of input configuration (*i*_*1*_, ⋯, *i*_*k*_) is thus determined through the hierarchy *o* = *O*_*1*_ if *i*_*1*_ = *I*_*1*_; *o* = *O*_*2*_ if *i*_*1*_ ≠ *I*_*1*_ and *i*_*2*_ = *I*_*2*_; ⋯; *o* = *O*_*k*_ if *i*_*1*_ ≠ *I*_*1*_, ⋯, *i*_*k* − *1*_ ≠ *I*_*k* − *1*_, *i*_*k*_ = *I*_*k*_; *o* = *NOT O*_*k*_ if *i*_*1*_ ≠ *I*_*1*_, ⋯, *i*_*k* − *1*_ ≠ *I*_*k* − *1*_, *i*_*k*_ ≠ *I*_*k*_.) We have successfully tested and validated the two properties for the LDOI of each node in the generated networks. We also tested and validated the properties of the LDOI of node sets of size up to 3~7 depending on the specific network (as the complexity of testing the property grows faster than *N*^*k*^ for *k* << *N*, where *N* is the network size and *k* is the node set size).

With respect to testing the target control algorithm, we generate 50 random target sets with size 2 or 3 for each random network. We calculate the average number of generated solutions for each pair formed by a target set and a network. As shown in Table 1, the average number of solutions is significantly high, between 10 and 40 for ensembles with nested canalizing functions and between 25 and 70 for ensembles with effective Boolean functions. The dominance of the canalizing variables in determining certain outcomes tends to yield sparser expanded networks than non-canalizing functions, and fewer effective interventions. This is reflected in the smaller number of solutions in the ensembles with nested canalizing functions compared to the ensembles with effective Boolean functions. As shown in Figure S1, the average time for finding solutions for a target set (through 500 iterations) is 100 s or less for networks with 15–35 nodes and 20–60 edges. As expected, the runtime increases with the number of nodes and edges, reaching 600 s for 50 nodes and 100 edges. The relatively slow increase and practical runtime suggest that our algorithm is effective for logical models of biological systems.

It is not always possible to find a solution for a specific target set for a network, especially when the Boolean network model does not have a (partial) fixed point type of attractor (i.e., if all nodes oscillate in the attractor) or when the desired target state set consists of node states that are part of different attractors, which conflict with each other. Another case where target control is impossible is when the target set is not reachable from the rest of the network. In the simulations of the two ensembles mentioned above, we verified that we are able to find a solution for more than 99.5% of the target sets when the target set satisfies two criteria: (i) it is a subset of a (partial) fixed point and (ii) the targets in this set are accessible from nodes outside of this set in the original network (that is, the targets do not consist of source nodes only and do not form a motif without any incoming edges). Note that there can be counter-examples where satisfying these criteria is not sufficient to find a solution. For example, in Figures 2A,B, there are no solutions for the target set {~*B*, ~*C*} as the remaining nodes are not enough to activate the composite node in Figure 2B. However, the probability of such situations is small in both random ensembles with moderate size and real biological network. Moreover, the fact that one cannot find a solution through our GRASP algorithm for the target control problem often indicates that the target set is not a reasonable target. It is likely that one would not be able to find a solution in such situation even with a whole state space search.

We also test the performance of different heuristic functions for the target control problem. We calculate the average number of generated solutions for each pair formed by a target set and a network. As shown in Table 1, greedy functions with a penalty for containing the negation of a node state included in the target set (score index 4 and 5) consistently perform better than the greedy functions directly using the size of the LDOI (score index 1 and 3). The intuition behind this is that it is more efficient to choose from those nodes whose DOI does not contain a conflict with the target. The second greedy function (|*Comp*_*LDOI*|) also performs quite well.

### 3.2. Biological examples

We applied our methodology on four Boolean models of signal transduction networks. The four Boolean models are freely available on GitHub (https://github.com/yanggangthu/BooleanDOI) in SBML Qual format and in our custom format. In the following we demonstrate our algorithm on two of these, the epithelial-to-mesenchymal transition (EMT) network and the PI3K mutant ER+ breast cancer network. The results on the ABA induced stomatal closure network and the T-LGL leukemia network are shown in Supplementary Materials sections 3.3, 3.4. Table 2 summarizes representative interventions found with our algorithm and compares them to the results of the most relevant previous analysis of these four biological network models. In Supplementary Data Sheet 1 we include the LDOI of each single node in the four models analyzed.

#### 3.2.1. EMT network

**Figure 3:**
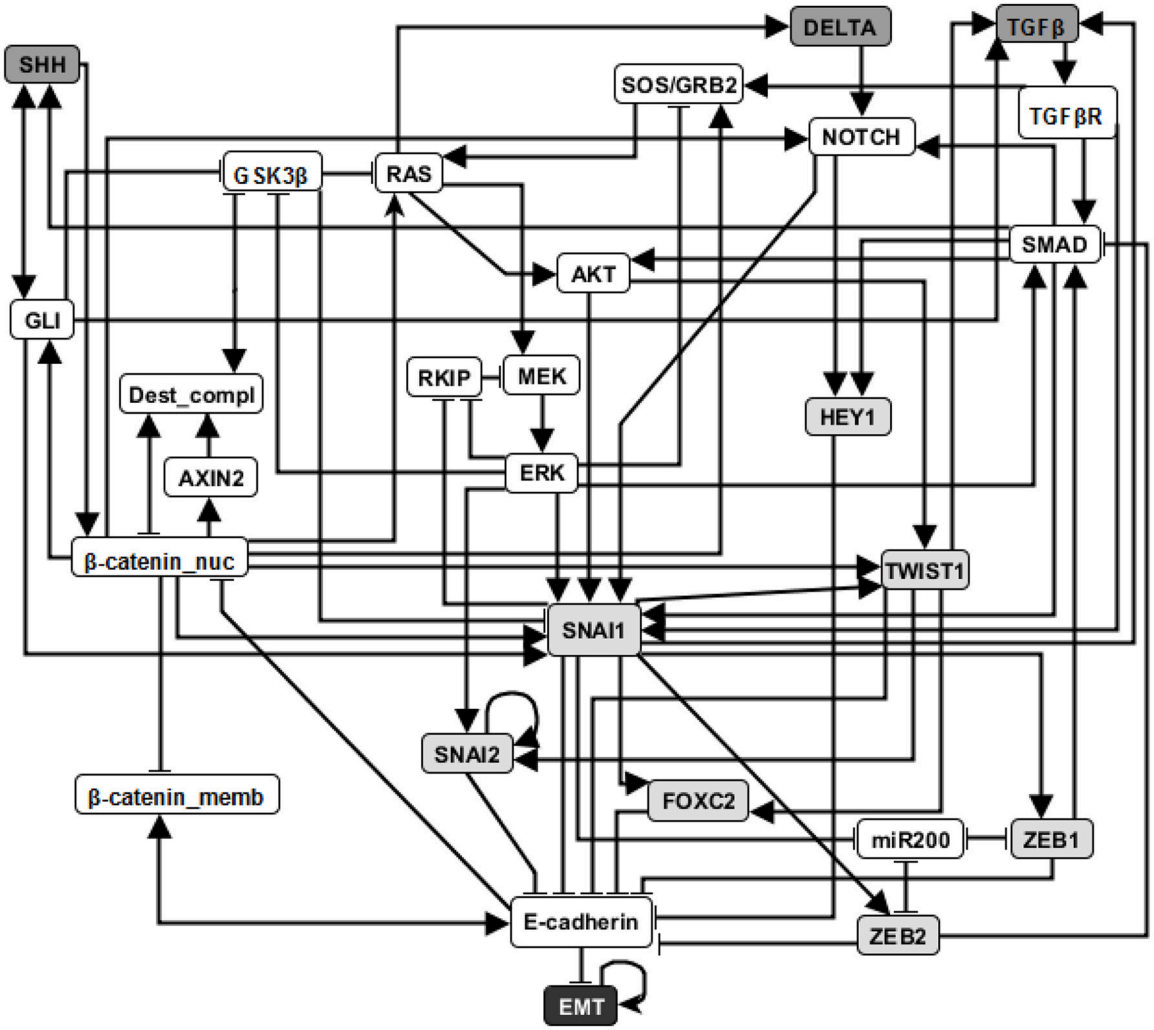
An illustration of the EMT network. Attractor-preserving network reduction (Saadatpour et al., 2013) was applied to better focus attention on the most relevant nodes. Specifically, source nodes that represent input signals that are absent in the studied context are removed and each node with one input or one output is absorbed into its input or output, respectively. Nodes with light gray background are direct regulators of E-cadherin and nodes with dark gray background represent external signaling molecules. Edges ending with an arrow represent positive regulation while edges end with a flat bar represent negative regulation. See more details in Supplementary Material section 3.1.

EMT is a cell fate change involved in embryonic development, which can be reactivated during cancer metastasis (Steinway et al., 2014). During EMT, epithelial cells lose their original adhesive property, and become mesenchymal cells which leave their primary site, invade neighboring tissue, and migrate to distant sites. A Boolean network model of EMT in the context of hepatocellular carcinoma invasion has been established by Steinway et al. (2014). Several predictions of this model were validated experimentally (Steinway et al., 2014, 2015). The EMT network has 70 nodes and 135 edges. The adhesion factor E-cadherin is the sink node; its OFF state indicates the transition to a mesenchymal state. The network has a normal (epithelial) steady state and an abnormal (mesenchymal) steady state. (See details in Supplementary Materials section 3.1). In Figure 3 we show a simplified version of the EMT network; our analyses were done on the full network.

Previous research on this network has indicated that sustained activation of TGFβ signal can trigger EMT through the activation of eight stable motifs (Steinway et al., 2015). In addition, stabilization of any of these stable motifs can drive EMT. Our analysis of the LDOI of each node state indicates that any of 60 node states (out of 138 node states for the 69 nodes) can lead to EMT, including the previously established EMT drivers. Moreover, 43 node states (nodes of the expanded network) have the same LDOI, which contains 48 node states, including EMT = 1 (see Supplementary Data Sheet 1). Each of these 43 node states is either the core of one or more of the eight stable motifs, or an external driver of one or more of the eight stable motifs. Thus the EMT outcome and the mesenchymal steady state has a large basin of attraction. As we are more interested in designing therapeutic strategies to block the epithelial to mesenchymal transition, we set the negation of EMT as a target. Previous analysis indicated that when considering an initial epithelial state and turning on the TGFβ signal, the knockout of any of the transcription factors that downregulate E-cadherin (i.e., knockout of SNAI1, SNAI2, FOXC2, TWIST1, ZEB1, ZEB2, HEY1) or multiple double node knockout combinations (knockout of SMAD and one of RAS, CSL, DELTA, NOTCH, NOTCH_ic, SOS/GRB2) are effective in blocking EMT (i.e., leading to E-cadherin = ON). The effectiveness of transcription factor knockout had been established in the literature; unfortunately these transcription factors cannot be targeted with existing drugs. Several double knockout combinations were validated experimentally in (Steinway et al., 2015) and are more amenable to drug targeting.

For EMT as target, our target control algorithm gives 7 two-node solutions (activation of β-catenin_memb and knockout of any of SNAI1, SNAI2, FOXC2, TWIST1, ZEB1, ZEB2, HEY1) and 5 three-node solutions (activation of β-catenin_memb, knockout of SMAD and knockout of any of RAS, CSL, DELTA, NOTCH, and NOTCH_ic). The main difference between the target control solution and the previously found EMT-blocking single and double knockout interventions is that our target control solution includes the additional control of β-catenin_memb. To understand this difference, we note that EMT is in the LDOI of TFGβ, however, EMT is not in the LDOI of the set consisting of TGFβ together with any of the previously found EMT-blocking knockout interventions. This indicates that the knockout intervention is effective in the sense that it can block the process of reaching EMT. However, ~EMT is also not in the LDOI of the set of TGFβ together with any knockout intervention. The knockout intervention is effective when the initial condition is the epithelial steady state, however the knockout intervention does not block EMT for all initial conditions. The target control algorithm, which can block EMT for all initial conditions, requires one more node (β-catenin_memb) in the target control solution. In fact, treating this problem as a damage mitigation problem, where the damage is sustained activation of TGFβ, we verify that EMT is in the LDOI of TGFβ together with any of the target control solutions.

As established in previous results, the single node EMT-blocking knockouts do not lead back to an epithelial state but rather to hybrid epithelial or mesenchymal steady states (Steinway et al., 2015). The hybrid epithelial steady state has certain epithelial features, e.g., E-cadherin and β-catenin_memb are activated, and also some mesenchymal features, e.g., MEK, ERK, and SNAI1 are activated. The hybrid mesenchymal steady state demonstrates the opposite features compared to the epithelial steady state. A good target set to avoid reaching such a hybrid state (which is likely pathological and may even be a worse outcome as the mesenchymal state) would be {~EMT, ~MEK} (Steinway et al., 2015). The minimum solution found involves controlling three nodes: activation of β-catenin_memb, inhibition of SNAI1, inhibition of RAS or RAF. We also find a four-node intervention that does not involve ERK and SNAI1: activation of β-catenin_memb, miR200 and RKIP, and also inhibition of RAS. If the target set is {~EMT, ~MEK, ~SNAI1}, the minimum solution size is found to be six.

Stable motif control indicates that control of at least five nodes is needed to drive any initial state (including the mesenchymal state) to the epithelial state (see Supplementary Table 3 of Steinway et al., 2015) Although the control goal is different, one can still see the connection between our target control solution for the target ~EMT and the stable motif control solution (to drive the system to the epithelial state). Specifically, they both require activation of β-catenin_memb. Knockout of SNAI1 , knockout of TWIST1, or knockout of SMAD and RAS, as one of the target control solutions, also appear as a part of stable motif control solution that does not require control of TGFβ or TGFβR.

These results demonstrate both the accuracy and effectiveness of our target control algorithm. The solutions found through 1,000 iterations are comprehensive (comparable to the solution found through a systematic search of knockout pairs). Our algorithm indicates intervention sites that are close to the target but also sites that are further away (e.g., SMAD). This diversity enables the selection of the most practical interventions.

#### 3.2.2. Breast cancer network

**Figure 4:**
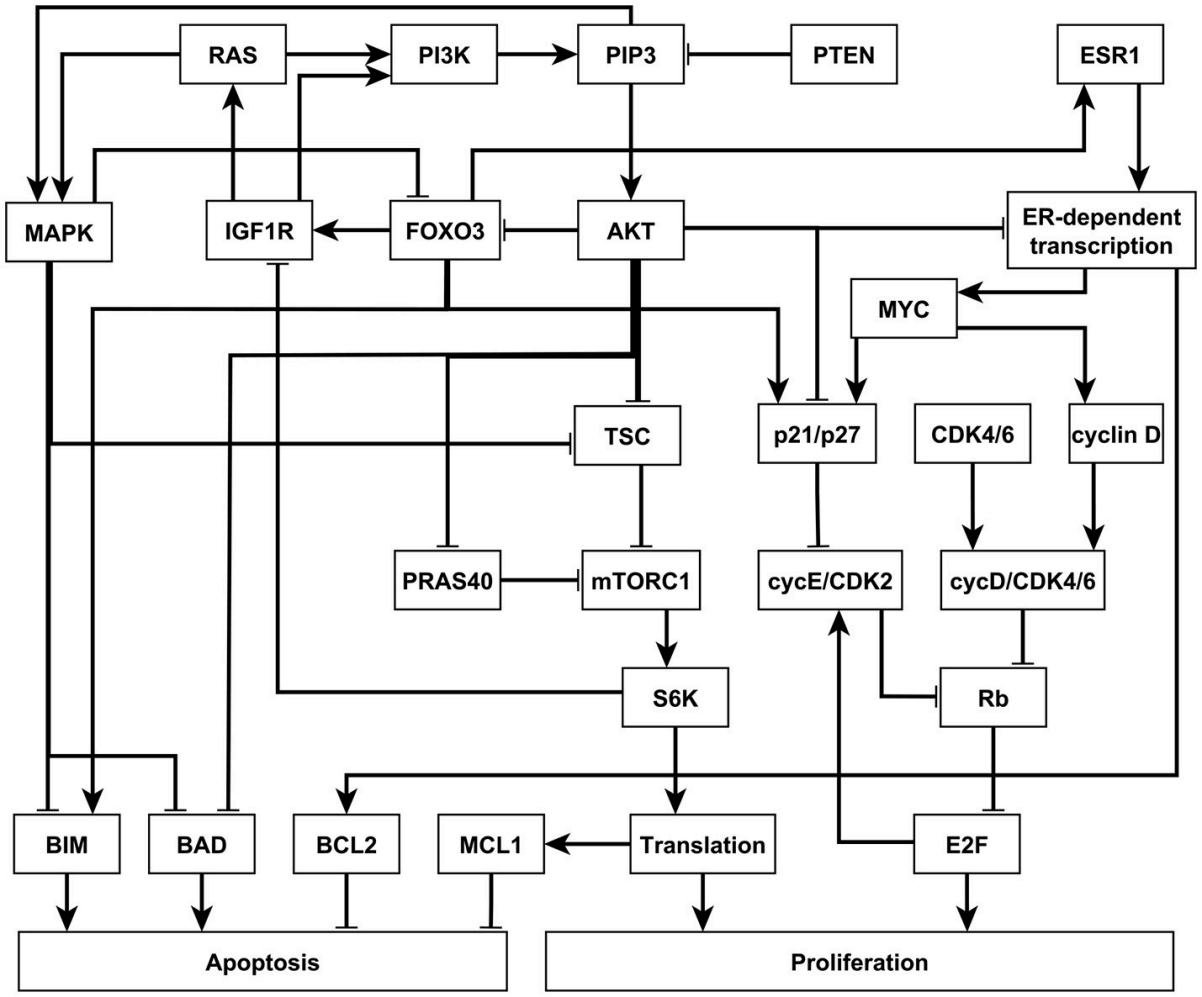
An illustration of the PI3K mutant, ER+ breast cancer network. Attractor-preserving network reduction was applied to focus on the nodes most relevant to our analysis. Nodes are colored according to the signaling pathway that they participate in. Edges ending with an arrow represent positive regulation while edges ending with a hollow diamond represent negative regulation. See more details in Supplementary Material section 3.2.

In 2017, Zañudo et al. established a discrete dynamical model of the signal transduction processes involved in the PI3K mutant, estrogen receptor positive (ER+) breast cancer, as shown in Figure 4 (Gómez Tejeda Zañudo et al., 2017). The model includes 58 nodes, which correspond to proteins, transcripts, drugs, and two cellular outcomes, apoptosis (programmed cell death) and proliferation (cell cycle progression). A fraction of the nodes (16), including the outcome nodes, are characterized by multiple levels, which is implemented by additional virtual nodes, e.g., apoptosis2 corresponds to level 2 of apoptosis, which has a more stringent regulatory function than apoptosis1 (level 1 of apoptosis). This network as implemented is essentially a Boolean network because all the regulatory functions are Boolean (Gómez Tejeda Zañudo et al., 2017). The network model successfully captures the key role of the PI3K/AKT/mTOR signaling pathway in determining the pathological proliferation and survival of cancer cells. In untreated simulated cancers cells, PI3K, MAPK, AKT, mTORC1, and ER signaling are active, leading to high level of proliferation and lack of apoptosis. The network model successfully captures the effectiveness of PI3K inhibiting drugs in leading to low level of proliferation and high level of apoptosis (Gómez Tejeda Zañudo et al., 2017). Through extensive simulations, the network model confirms known drug resistance mechanisms, i.e., additional mutations or other dysregulations that lead to the loss of effectiveness of PI3K-inhibiting drugs. It also predicts new possible resistance mechanisms and the degree of survivability under different resistance mechanisms (Gómez Tejeda Zañudo et al., 2017).

Similar insights can be drawn by LDOI analysis and applying the target control algorithm to the discrete dynamical network model without doing dynamical simulations, which demonstrate the rich information contained in the network topology and logic and the effectiveness of our control methodology. We obtained a (relatively large) reduced network by considering the system under the relevant initial condition of PI3K mutant, ER+ cancerous state, while keeping the seven drugs as source nodes (see details in Supplementary Material section 3.2). The five node states with the highest LDOI are Fulvestrant, ~ESR1 (which both mean the inhibition of the estrogen receptor) and Alpelisib, ~PI3K, ~PIP3 (which all mean the inhibition of PI3K). The LDOI of these four nodes is very similar and includes 18 node states, including a high level of apoptosis (Apoptosis = 2), and a reduction in proliferation. Other drugs or node inhibitions yield subsets of the largest LDOI (see Supplementary Data Sheet 1). These results are consistent with, and yield further insight into the current knowledge on the effect of drugs in this network. If we now set the target to be high level of apoptosis and no proliferation, i.e., Target = {Apoptosis2, ~Proliferation}, the algorithm gives multiple two-node interventions as minimal interventions, these consists of either of {~PI3K, ~PIP3} and inhibition of any node in the MYC-CDK4/6 axis of cell-cycle regulation, i.e., {~ESR1, ~ER_transcription, ~MYC, ~CDK46, ~cyclinD, ~cycD_CDK46, ~Rb, ~E2F}. There are several drugs that can target these nodes. For example, Alpelisib is a PI3K inhibitor, Fulvestrant is a ESR1 inhibitor and Palbociclib is a CDK4/6 inhibitor. This result is consistent with the results found in the (Gómez Tejeda Zañudo et al., 2017): inhibition of PI3K leads to an increase in ER transcriptional regulatory activity, leading to a decrease in proliferation, and simultaneous PI3K and ER inhibition has a synergistic effect in completely blocking proliferation and maintaining a high level of apoptotic activity. If PI3K inhibitor or PIP3 inhibitor is not allowed to be used, the algorithm finds three node solutions involving an AKT inhibitor (e.g., Ipatasertib), MAPK inhibitor (e.g., Trametinib) and inhibition of any node from the MYC-CDK4/6 axis of cell-cycle regulation. In other words, inhibition of AKT together with MAPK provides a similar functionality with inhibition of PI3K. One can also use the LDOI to identify possible drug resistance mechanisms, i.e., perturbations that make PI3K inhibition less effective. As {Apoptosis2,~Proliferation4} ⊂ LD (~PI3K), we simply go through all possible two-node interventions containing PI3K inhibitor and screen out those interventions whose LDOI either does not contain Apoptosis2 or contain Proliferation3 or higher level (Proliferation4). We reproduce most of the potential drug resistance mechanism to PI3K inhibitors indicated in Table 3 of Gómez Tejeda Zañudo et al. (2017).

## 4. Discussion

In summary, we have developed the new measures DOI and LDOI to describe the long-term effect of a sustained intervention. We have applied these measures to find solutions to the target control problem in logical network models. This work takes a step forward toward practical control of real biological systems, as illustrated by the applications presented here. The target control solutions we find recover previous predicted interventions obtained by other methods (dynamic simulations and stable motif analysis). As several of these previous predictions are validated experimentally, this agreement also serves as validation of our target control solutions. Notably, by generating a large number of valid target control solutions, we are going significantly beyond previous results (see Table 2). The multitude of predicted target control interventions allows their filtering according to biological or technological considerations.

Here we assumed the existence of a discrete dynamical model. As there are significant uncertainties in the existing models due to the scarcity of experimental information, we estimate the sensitivity of the LDOI measure to the incompleteness of the dynamical model. As the primary way of obtaining causal information that can be used in a logical model is to perform knockout experiments, the predominant causal information indicates a node as being necessary for the activation of another node. For example, if the knockout of either of two regulators A or B leads to a decrease in the activity of target C, we would infer that the logical rule for C is *C* = *A*AND*B*. Suppose that there is a so far undetected regulator of C, which we denote by X. This X will likely also be necessary, which would maintain agreement with the previous observations, i.e., *C* = *A*AND*B*AND*X* is the true rule. Consider the rule for the complementary node ~*C* = ~*A* OR ~*B* in the case of the incomplete system versus the true rule ~*C* = ~*A* OR ~*B* OR ~*X*. We can see that the LDOI of any of ~*A*, ~*B, A, B* will be robust to the addition of X. The LDOI of node *X* and ~*X* need to be established in the true system. The LDOI of node state set {*A, B*} will be affected by this change. (However, LDOI of ~*A* and ~*B* will not change). Thus the size of the solution of the target control problem may increase due to this incomplete information. Due to the binary essence of the Boolean rule, missing a sufficient regulator (an extra OR rule) will give similar results.

The LDOI is closely related to previously introduced concepts and methods used to analyze Boolean models. In particular, the LDOI of a node state (or a set of node states) is mathematically equivalent to the three-valued logical steady state that results when these node states of interest are fixed (Klamt et al., 2006; Samaga et al., 2010) and is also equivalent to the set of node states that become stationary if using network reduction techniques after fixing the node(s) of interest in the appropriate state(s) (Bilke and Sjunnesson, 2001; Naldi et al., 2012; Saadatpour et al., 2013). The work presented here goes beyond previous work and identifies general properties of the LDOI of node states and their union (the first key property of the LDOI), and of the relation of the LDOI and stable motifs (the second key property of the LDOI).

The algorithm to identify the LDOI using the expanded network bears similarities with the algorithm in Samaga et al. (2010) and Klamt et al. (2006), which uses signed interaction hypergraphs to calculate logical steady states resulting from fixing node states. An important difference is that the expanded network assigns a complementary node to each node to denote the inactive state of a node, while the hypergraph representation instead assign signs to nodes and to composite nodes to keep track of their states. Although the LDOI obtained using either method is the same, we argue that the expanded network representation has several desirable properties that differentiate it, in particular, (i) it makes the interpretation of the LDOI more intuitive and the algorithm for calculating it purely graph-theoretical, i.e., a modified breadth-first search on the expanded network, (ii) it treats the active/inactive states equally (a reflection of the fact that a change of variables can redefine what an active/inactive state means), and (iii) it provides a natural way to generalize the LDOI from Boolean to discrete models by defining a virtual node for each allowed node state (e.g., if a node has 3 states we would have 3 virtual nodes: one denoting state 0, one denoting state 1, and one denoting state 2).

The DOI and LDOI is also related the concept of elementary signaling mode (ESM), originally defined as a minimal subgraph that can propagate a signal from a source node to an output node (Wang and Albert, 2011; Sun and Albert, 2016). An ESM on the expanded network is the generalization of a path on a usual directed network. Similarly, the LDOI of a node on the expanded network is analogous to a connected component reachable from a node on a usual directed network. In the same way a connected component reachable from node i consists of nodes that have a path starting from node *i*, the LDOI of a node consists of all the nodes included in any ESM that starts from that node. Recent work by Maheshwari and Albert (2017) developed a logic framework to identify causal relationships that are sufficient or necessary. This framework allows an alternative definition of the LDOI. The LDOI of the ON state of a node (${\tilde{\sigma }}_{i}=1$) includes all the nodes for which the node is a sufficient activator (these nodes will have ${\tilde{\sigma }}_{j}=1$) or sufficient inhibitor (these nodes will have ${\tilde{\sigma }}_{k}=0$). Similarly, the LDOI of the OFF state of a node includes all the nodes for which the node is a necessary activator (these nodes will have ${\tilde{\sigma }}_{j}=0$) or necessary inhibitor (these nodes will have ${\tilde{\sigma }}_{k}=1$).

An algorithm to construct ESMs through a backward search from an output node was presented in Wang et al. (2013); this algorithm can be adapted to find solutions of the target control problem of a single output. If we treat the output node as the root of a backward search, the set of nodes found in the ESM in each search depth (distance from the output node) can serve as a control solution. A truncation technique similar to ours needs to be applied to deal with inconsistent feed-forward or feed-back loops. This algorithm can be generalized to solve the target control problem of a target set by simultaneous search from each target node. We chose to transform the target control problem into a planning search problem; and it has been established that such a planning search problem can be solved in both a forward propagation and a backward propagation approach, or even a mixed approach (Russell and Norvig, 2003). It will be an interesting future work if such techniques can improve the efficiency of the algorithm.

This work points out interesting questions as future research directions. First, though evaluating the DOI of a node (set) is computationally hard, a better estimation of the DOI rather than the LDOI is desirable and can be used to reduce the size of the solution given by our current target control algorithm. Second, the requirement that the solution works for all initial conditions in the setup of the target control problem gives robust solutions, however it may be overly conservative for biological systems in certain applications, especially if one is certain about the relevant initial condition subspace. A semi-structural approach (without doing dynamical simulations) to solve the target control problem starting from a subspace of initial conditions are also desirable.

## Author contributions

GY, JG, and RA designed research and methodology. GY and RA performed the analyses. GY, JG, and RA wrote the paper.

### Conflict of interest statement

The authors declare that the research was conducted in the absence of any commercial or financial relationships that could be construed as a potential conflict of interest.
