# Supplementary material for: Target Control in Logical Models Using the Domain of Influence of Nodes
Source: Front Physiol. 2018 May 8;9:454. doi: 10.3389/fphys.2018.00454 (PMC5951947; doi:10.3389/fphys.2018.00454)
Supplement: Supplementary file 1 [file Presentation_1.pdf]

Supplementary Material: Target Control in Logical Models Using the Domain of Influence of Nodes

1 ALGORITHMS

1.1 Algorithm for calculating LDOI

Here we present the pseudocode for calculating the LDOI of a set of nodes, referred to as $source$, on the expanded network $G\_expanded$. $Negation(node)$ in line 8 calculates the negation of a non-composite $node$ on the expanded network. The $Children(G\_expanded, node)$ function in line 11 returns all direct neighbors that $node$ points to on the expanded network. The $InDegree$ function in line 14 calculates the in-degree of a node on the expanded network (number of incoming edges or number of parent nodes).

Algorithm 1 Algorithm of calculating LDOI

```
1:  procedure LDOI(G_expanded, source)
2:      queue ← Queue(source)
3:      visited ← Set()
4:      visited_list ← List()
5:      cpnode_count ← HashMap()
6:      while queue is not empty do
7:          node ← queue.pop()
8:          if node ∉ visited AND Negation(node) ∉ visited then
9:              visited ← visited ∪ {node}
10:             visited_list.append(node)
11:             for child in Children(G_expanded, node) do
12:                 if child is not composite node then
13:                     queue.append(child)
14:                 else if cpnode_count[child] = G_expanded.InDegree(child) − 1 then
15:                     queue.append(child)
16:                 else
17:                     cpnode_count[child] += 1
18:                 end if
19:             end for
20:         else if node ∈ source then
21:             visited_list.append(node)
22:         end if
23:     end while
24:     return Set(visited_list[length(source) : ])
25: end procedure
```

1.2 Algorithm of the local search for solution reduction

Here we give the pseudocode to obtain a reduced solution from a candidate solution, referred to as $solution$, in the target control problem on the expanded network $G\_expanded$ with $Target$ as the target.

Algorithm 2 Algorithm for local search of solution reduction

```
procedure LocalSearch(G_expanded, Target, solution)
    reduced_solution ← solution
    if solution.length() ≤ 1 then
        return reduced_solution
    end if
    for node in random order of solution do
        temp_solution ← reduced_solution
        temp_solution.remove(node)
        if Target ⊂ LDOI(solution) then
            reduced_solution ← temp_solution
        end if
    end for
    return reduced_solution
end procedure
```

1.3 Algorithm of the MakeRCL function

Here we present the pseudocode to construct a restricted candidate list (RCL) from candidates given the greedy function $G(v)$ and random number α.

Algorithm 3 Algorithm of the MakeRCL function

```
procedure MakeRCL(candidates, G(v), α)
    candidate_scores ← {G(v)|v ∈ candidates}
    G_max ← max(candidates_scores)
    G_min ← min(candidates_scores)
    G_pass ← G_min + α · (G_max − G_min)
    RCL ← {v ∈ candidates|G(v) ≥ G_pass}
    return RCL
end procedure
```

A python implementation of the target control algorithm is available at

https://github.com/yanggangthu/BooleanDOI.

2 DEFINITION AND PROPERTIES OF THE LDOI

2.1 Mathematical description of the level-order preserving updating regime

Mathematically, let s^{ex} be the node in the expanded network whose logical domain of influence we are seeking to determine, and s the corresponding node in the original network. Let $d(s, i)$ represent the distance (shortest path length) from s^{ex} to n_i or $\sim n_i$ on the expanded network. $d(s, s) = 0$ when there is no feedback loop from node s to node s, otherwise, $d(s, s) = l_s$, which is the length of the shortest feedback loop passing s. Let t_i be the first time node i is updated. In a level order preserving updating regime, $t_i \leq t_j$ if and only if $d(s, i) \leq d(s, j)$.

2.2 Mathematical proof of the second key property of LDOI

We prove the property by contradiction. The property is: if the LDOI of a node state set S contains itself, then the LDOI of S contains a stable motif. The contradiction statement is that $S \subset \mathcal{LD}(S)$ implies

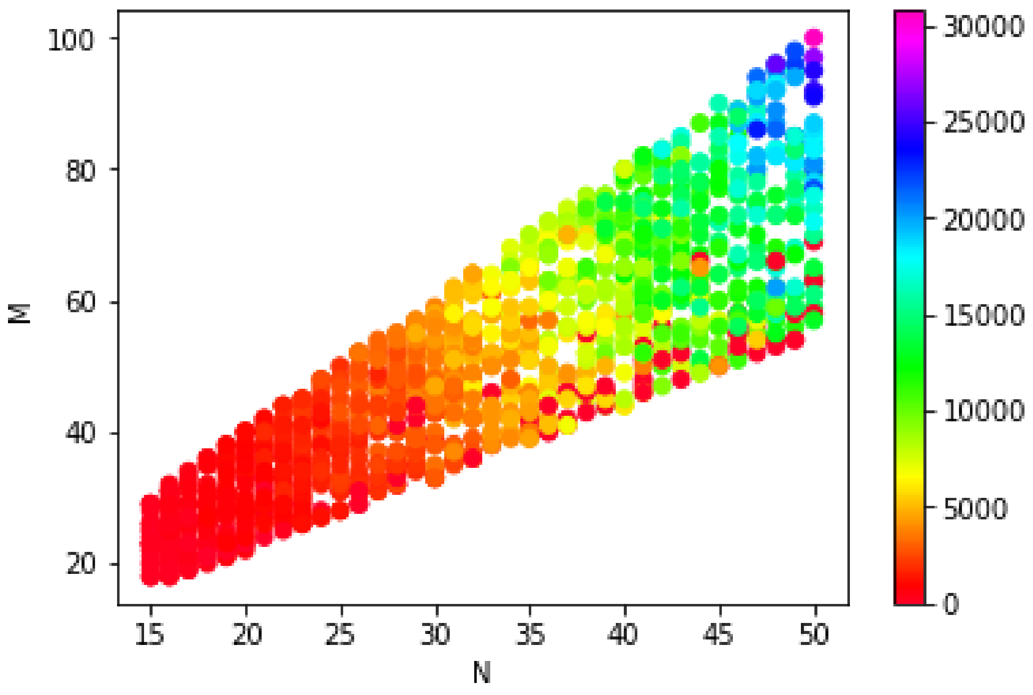

Figure S1: Running time versus number of nodes and edges of a network.

that $\mathcal{LD}(S)$ does not contain a stable motif. $S \subset \mathcal{LD}(S)$ implies that there exists a level order preserving updating regime in which node states in S will not change when these nodes are updated and are not externally controlled (fixed). This level order preserving updating regime can be constructed by periodically repeating the first round of updating. In such an updating regime, the system will evolve into a (partial) fixed point as node states in S does not change. However, in the contradiction statement, $\mathcal{LD}(S)$ does not contain a stable motif. Thus no subset of nodes belonging to S can be a (partial) fixed point since a stable motif corresponds to a (partial) fixed point (proved in Zañudo and Albert (2013)), where we find the contradiction. Thus the contradiction statement is false and the original property holds.

3 ADDITION INFORMATION ON RANDOM BOOLEAN NETWORKS

In Fig. S1 we plot the run time in seconds (represented by the color) as a function of the number of nodes N (horizontal axis) and the number of edges M (vertical axis). The run time is the total duration of considering 50 randomly selected target sets (of two and three targets), with solutions for each target set calculated in 500 individual iterations in a serial mode (*i.e.* $max\_itr = 500$ for line 4 in Algorithm Table 1) for the ensemble with nested canalizing functions.

4 ADDITIONAL INFORMATION ON THE BIOLOGICAL EXAMPLES

4.1 EMT network

Detailed information about the EMT network can be found in Steinway et al. (2014, 2015). The Boolean regulatory functions are presented in the Supplemental Table 1 of Steinway et al. (2015). The two steady

states of the EMT network model are shown in Supplemental Table 2 of Steinway et al. (2015). Previous knockout intervention results are illustrated in Figure 1 of Steinway et al. (2015).

4.2 Breast cancer network

Detailed information about the breast cancer network, including the Boolean regulatory functions of each node, can be found in the section "Regulatory functions in the model" of the additional file in Gómez Tejeda Zañudo et al. (2017). Relevant initial conditions that lead to cancerous steady states, which have slight variations among themselves, are stated in the section "Initial or externally controlled states" of the additional file in Gómez Tejeda Zañudo et al. (2017). For our purpose of target control, we chose the initial condition that leads to the most cancerous steady state and obtained a (relatively large) reduced network after plugging in these initial conditions. Specifically, among the 12 source nodes, IGFR1_T, PBX1 and ER are fixed to be ON. HER2, HER3_T, PTEN, SGK1_T, PIM, PDK1 and mTORC2 are fixed to be OFF. BIM_T is assumed to be OFF while BCL2_T is assumed to be ON. The seven source nodes corresponding to the drugs are still kept as source nodes in the network. The outcome nodes "Apoptosis" and "Proliferation" are multi-level nodes representing a cell′s propensity to commit programmed cell death or cell cycle progression, respectively. Apoptosis has four levels and is represented by three Boolean nodes. Proliferation has five levels and is represented by four Boolean nodes. The highest level of apoptosis corresponds to full commitment to apoptosis and lower levels correspond to partial commitment. The regulatory function that represents a higher level of apoptosis or proliferation is more stringent compared to the regulatory function of a lower level. Under the current chosen initial condition and without any drugs, the natural attractors are cancerous steady states with low level of apoptosis (Apoptosis = 0) and high level of proliferation (Proliferation = 3 or 4). In the reduced network under the currently chosen initial condition, PI3K inhibitor can only lead to apoptosis level 2 and no drug (combinations) can lead to apoptosis level 3.

4.3 ABA induced stomatal closure network

On the epidermes of leaves and other aerial plant parts, a pair of guard cells modulate the size of natural openings known as stomata, which are the entry and exit points of gas exchange. Albert et al. constructed a Boolean model of the signaling process involved in stomatal closure in response to the drought hormone abscisic acid (ABA) Albert et al. (2017). The nodes of the network include signaling proteins, small molecules, effectors of ion flow and conceptual nodes such as the outcome node "Closure". The detailed Boolean rules are given in Table 2 and S1 Text of Albert et al. (2017). The relevant initial conditions (which specify the state of 54 nodes based on biological knowledge and leave 26 unspecified) and corresponding natural attractors are given in Table S6 and S7 of Albert et al. (2017).The dynamic model captures the process of stomatal closure in response to ABA and the robustness of this outcome to variations in the initial conditions. Albert et al. also systematically studied the effect of knockout and constitutive activity in the presence or absence of ABA, obtaining many results consistent with prior experimental results. Two model predictions were tested and validated experimentally in Albert et al. (2017). The model predicts that in the absence of ABA, only the constitutive activation of reactive oxygen species (ROS) will lead to a high closure probability similar to the case in the presence of ABA.

We first ask what nodes need to be controlled to lead to closure in the absence of ABA. Inspecting the LDOI of each node state (Supplemental Data Sheet S1), we find that there are no node states that have Closure in their LDOI. Conversely, the lack of closure ($\sim$Closure) is in the LDOI of 19 node states, one of which represents the activity of the inhibitory protein ABI1 and the rest represent the inactive states of proteins such as OST1, SphK1/2, RBOH and low concentrations of small molecules such as PA, ROS. The LDOI of these latter proteins contains the stable motif previously identified to correspond to the absence of

closure in the absence of ABA (see Fig. **2E** in Albert *et al.* 2017). Thus each of these 18 node states is either a core, or an external driver of this stable motif. The largest LDOIs are those of Ca_c^{2+}, ROS and RBOH (around 20 node states), but they do not contain Closure or its direct regulators. Turning to the target control algorithm to answer the question, the minimal solution given by our algorithm involves at least two nodes. There are only two compatible solutions with size two, that is, activation of H_2O efflux and Microtubule or TCTP. This solution is not very insightful as these nodes are direct regulators of the node Closure. The algorithm gives several incompatible solutions: Ca^{2+} and any of ROS, RBOH, OST1, ∼ABI2, ∼HAB1, ∼PP2CA or H_2O efflux. The solutions are incompatible as Ca^{2+} is regulated by a negative feedback loop and oscillates in the natural dynamics of the system. The fact that the target control solution requires more than activation of ROS is due to the fact that the target control solution is applicable for all initial conditions, while the knockout or constitutive activation study starts with biologically relevant initial conditions.

Another interesting target is the activation of H_2O efflux, a key contributor (and upstream node) of stomatal closure. The incompatible solutions indicated above still hold (except Ca^{2+} and H_2O efflux since H_2O efflux is the target). The minimal compatible solutions involve three nodes, as shown in Table S1. Interestingly, the LDOI of these solutions do not contain closure, meaning that they are enough to activate H_2O efflux but not enough to activate closure.

Table S1. The minimal compatible solutions for activating H_2O efflux. The three-node solution comprises of the activation of any node from each column in the same row.

A	B	C
Kefflux	AnionEM, SLAC1	ROS, RBOH, OST1, PIP21
VATPase, Vacidification	AnionEM, SLAC1, MAPK912	ROS, RBOH

4.4 T-LGL leukemia network

T cell large granular lymphocyte (T-LGL) leukemia is a rare blood cancer, where leukemic T-LGL cells do not undergo activation induced cell death (apoptosis) after successfully fighting a virus like a normal T cells do. Zhang et al. (2008) construct a network model consisting of the proteins involved in the activation of T cells, in activation induced cell death, as well as a number of proteins that were observed to be abnormally highly expressed or active in T-LGL cells. The original network has 60 nodes and 142 regulatory edges, details of which and the regulatory functions can be found in Tables S1-S4 of Zhang et al. (2008). The model captures the normal (apoptosis) and leukemic (survival) states of the system Zhang et al. (2008); Saadatpour et al. (2011). For the discussion below, we consider the biological relevant initial condition where the source nodes IL15, Stimuli are ON and PDGF, Stimuli2, CD45 and TAX are OFF and the cell is in a resting (inactive) state.

Previous, experimentally validated research established that S1P = 0, PDGFR = 0, SPHK1 = 0 forms a stable motif and fixing any of the three nodes in the OFF state can control the stable motif, which can drive the system to the normal steady state, *i.e.* Apoptosis. Indeed, the LDOI of the three complementary nodes is identical and contains all three of them as well as three other nodes. However, these LDOIs do not contain the node Apoptosis. Another group of 6 nodes, namely IL2RB, IL2RBT, RAS, GRB2, ZAP70, PLCG1 also have identical LDOIs of 32 node states, which correspond to another previously identified stable motif of this network (the blue motif in Fig. 3 of $Za\tilde{n}udo\ and\ Albert$ 2015). The target control

algorithm′s minimal solutions for the target Apoptosis involve control of two nodes, namely any of ∼S1P, ∼SPHK1, ∼PDGFR combined with any of IL2RB, IL2RBT, RAS, GRB2, PI3K, NFκB. This discrepancy illustrates the difference between LDOI and DOI. The LDOI of neither stable motif contains Apoptosis. However, the stable motif analysis of *Zañudo and Albert* (2015) indicates that once the ∼S1P, ∼PDGFR, ∼SPHK1 stable motif is established, the system′s natural dynamics will lead to the stabilization of the other motif and finally will converge to Apoptosis. Thus in this case our target control algorithm gives a solution that is more stringent than necessary, which can guarantee to reach apoptosis based solely on the logical regulatory functions. When combined with stable motif control, we could do a target control search of the activation stable motif and find alternative ways to reach the desired state without directly controlling the stable motif.

REFERENCES

Zañudo JGT, Albert R. An effective network reduction approach to find the dynamical repertoire of discrete dynamic networks. *Chaos: An Interdisciplinary Journal of Nonlinear Science* **23** (2013) 025111. doi:10.1063/1.4809777.

Steinway SN, Zañudo JG, Ding W, Rountree CB, Feith DJ, Loughran TP, et al. Network modeling of TGFβ signaling in Hepatocellular Carcinoma Epithelial-to-Mesenchymal Transition reveals joint sonic Hedgehog and Wnt pathway activation. *Cancer Research* **74** (2014) 5963–5977. doi:10.1158/0008-5472.CAN-14-0225.

Steinway SN, Zañudo JGT, Michel PJ, Feith DJ, Loughran TP, Albert R. Combinatorial interventions inhibit tgfb-driven epithelial-to-mesenchymal transition and support hybrid cellular phenotypes. *Npj Systems Biology And Applications* **1** (2015) 15014 EP –. Article.

Gómez Tejeda Zañudo J, Scaltriti M, Albert R. A network modeling approach to elucidate drug resistance mechanisms and predict combinatorial drug treatments in breast cancer. *Cancer Convergence* **1** (2017) 5. doi:10.1186/s41236-017-0007-6.

Albert R, Acharya BR, Jeon BW, Zañudo JGT, Zhu M, Osman K, et al. A new discrete dynamic model of aba-induced stomatal closure predicts key feedback loops. *PLOS Biology* **15** (2017) 1–35. doi:10.1371/journal.pbio.2003451.

Zhang R, Shah MV, Yang J, Nyland SB, Liu X, Yun JK, et al. Network model of survival signaling in large granular lymphocyte leukemia. *Proc. Natl. Acad. Sci. USA* **105** (2008) 16308–16313.

Saadatpour A, Wang RS, Liao A, Liu X, Loughran TP, Albert I, et al. Dynamical and structural analysis of a T cell survival network identifies novel candidate therapeutic targets for large granular lymphocyte leukemia. *PLoS Computational Biology* **7** (2011) e1002267.
